# Postmortem metabolomics: influence of time since death on the level of endogenous compounds in human femoral blood. Necessary to be considered in metabolome study planning?

**DOI:** 10.1007/s11306-024-02117-y

**Published:** 2024-05-09

**Authors:** Andrea E. Steuer, Yannick Wartmann, Rena Schellenberg, Dylan Mantinieks, Linda L. Glowacki, Dimitri Gerostamoulos, Thomas Kraemer, Lana Brockbals

**Affiliations:** 1 Department of Forensic Pharmacology and Toxicology, Zurich Institute of Forensic Medicine, University of Zurich, Winterthurerstrasse 190/52, 8057 Zurich, Switzerland; 2 Centre for Forensic Science, School of Mathematical and Physical Sciences, Faculty of Science, University of Technology Sydney, Sydney, Australia; 3 Department of Forensic Medicine, Monash University, Victoria, Australia; 4 Victorian Institute of Forensic Medicine, Victoria, Australia

**Keywords:** PMR, Metabolomics, Carnitine, Amino acid, Time-dependent change

## Abstract

**Introduction:**

The (un)targeted analysis of endogenous compounds has gained interest in the field of forensic postmortem investigations. The blood metabolome is influenced by many factors, and postmortem specimens are considered particularly challenging due to unpredictable decomposition processes.

**Objectives:**

This study aimed to systematically investigate the influence of the time since death on endogenous compounds and its relevance in designing postmortem metabolome studies.

**Methods:**

Femoral blood samples of 427 authentic postmortem cases, were collected at two time points after death (854 samples in total; t1: admission to the institute, 1.3–290 h; t2: autopsy, 11–478 h; median ∆*t* = 71 h). All samples were analyzed using an untargeted metabolome approach, and peak areas were determined for 38 compounds (acylcarnitines, amino acids, phospholipids, and others). Differences between t2 and t1 were assessed by Wilcoxon signed-ranked test (*p* < 0.05). Moreover, all samples (*n* = 854) were binned into time groups (6 h, 12 h, or 24 h intervals) and compared by Kruskal–Wallis/Dunn’s multiple comparison tests (*p* < 0.05 each) to investigate the effect of the estimated time since death.

**Results:**

Except for serine, threonine, and PC 34:1, all tested analytes revealed statistically significant changes between t1 and t2 (highest median increase 166%). Unpaired analysis of all 854 blood samples in-between groups indicated similar results. Significant differences were typically observed between blood samples collected within the first and later than 48 h after death, respectively.

**Conclusions:**

To improve the consistency of comprehensive data evaluation in postmortem metabolome studies, it seems advisable to only include specimens collected within the first 2 days after death.

**Supplementary Information:**

The online version contains supplementary material available at 10.1007/s11306-024-02117-y.

## Introduction

Metabolomics (metabolic profiling) aims to comprehensively analyze endogenous low molecular weight compounds within biological systems, (e.g., amino acids and lipids). It represents the downstream output of the -omics cascade (genomics, transcriptomics, proteomics/peptidomics) and is also highly influenced by environmental factors, such as lifestyle habits, diseases, and drug intake. Over the years, a number of metabolomics techniques have been established in a variety of disciplines for biomarker search or for generating hypotheses, as different environmental stimuli may lead to particular changes within the metabolome (Castillo-Peinado & Luque de Castro, 2016; Johnson et al., 2016; Patti et al., 2012; Steuer et al., 2019, 2021; Wishart, 2016; Zeki et al., 2020). In this regard, untargeted metabolome acquisition approaches which theoretically measure all compounds simultaneously are used. Data acquisition is followed by sophisticated data evaluation strategies and statistical methods to identify compounds of interest. Depending on the data set and underlying question, simple univariate statistics, i.e., (non)parametric significance testing in combination with fold-change analysis, as well as multivariate statistics for multifactorial phenomena, or holistic models applying machine learning algorithms can be applied (Anwardeen et al., 2023; Chen et al., 2023; Pomyen et al., 2020; Procopio et al., 2023). Recently, the (un)targeted analysis of endogenous compounds has also gained interest in the field of forensic postmortem investigations, e.g., for assessment of biomarkers of the postmortem interval (PMI) (Bonicelli et al., 2022; Chighine et al., 2023; Donaldson & Lamont, 2013, 2014; Locci et al., 2019, 2021; Mora-Ortiz et al., 2019; Pesko et al., 2020; Peyron et al., 2019), postmortem redistribution (PMR) (Brockbals et al., 2020, 2021), or the improved interpretation of the cause of death (COD) (Cao et al., 2023; Elmsjo et al., 2021, 2022; Nariai et al., 2022; Ward et al., 2022).

However, the highly dynamic nature of the metabolome needs to be considered during the study design to allow observed effects to be attributable to the research question. Postmortem specimens are considered even more challenging as death is a dynamic process in itself, which introduces other, unpredictable variations. From numerous investigations and routine experience, the phenomenon of PMR is well recognized in forensic toxicology. PMR refers to all artificial changes in the postmortem concentrations of drugs after death (Pelissier-Alicot et al., 2003; Skopp, 2004, 2010). While certainly not fully understood, passive diffusion, degradation, or drug neo-formation represent the most common underlying mechanisms, as do factors such as the drug properties (lipophilicity, protein binding affinity, volume of distribution, basicity). Both ante- and postmortem biochemical processes also play a role (Drummer & Gerostamoulos, 2023; Peters & Steuer, 2018). Recent studies suggest the COD, manner of death (Elmsjo et al., 2021, 2022; Ward et al., 2022) and, the PMI between death and sample collection (Bonicelli et al., 2022; Chighine et al., 2023; Donaldson & Lamont, 2013, 2014; Locci et al., 2019, 2021; Mora-Ortiz et al., 2019; Pesko et al., 2020; Peyron et al., 2019) contribute to the postmortem metabolome composition. For instance, decreased levels of short-, medium- and long-chain acylcarnitines in human blood were observed to be related to oxycodone intoxication (Elmsjo et al., 2022). Elmsjo et al., reported higher concentrations of cortisol, phenylacetylglutamine, valerylcarnitine, or phenylalanine, or decreased concentrations of palmitoylcarnitine and various lysophosphatidylcholines in blood samples were associated with deaths attributed to pneumonia relative to a control group (Elmsjo et al., 2021). Previous studies also investigate the potential of using PMI-dependent concentration changes of endogenous molecules for biochemical estimation of the time of death. A variety of endogenous compounds were shown to increase with time, including different amino acids (hydroxyproline, tyrosine, phenylalanine), creatinine, citrate cycle intermediates (α-ketoglutarate, succinate), lactate, niacinamide, taurine, or uracil (Donaldson & Lamont, 2014; Du et al., 2018; Mora-Ortiz et al., 2019; Pesko et al., 2020). In contrast to human forensic investigations, where femoral blood or serum are the most commonly used matrix most studies on PMI estimation were performed in either animal models and/or specimens other than femoral blood (Chighine et al., 2023; Donaldson & Lamont, 2014; Du et al., 2018; Locci et al., 2019, 2021; Mora-Ortiz et al., 2019; Pesko et al., 2020); that said, there is still a lack of comprehensive studies with sufficient case numbers (Zelentsova et al., 2020). According to a recent publication, PMI can be considered the main driving force of postmortem metabolome changes, highlighting the need for more data and standardization for postmortem metabolomics studies that aim to answer research questions other than assessing the PMI (Chighine et al., 2021).

Our current study aimed to comprehensively investigate the influence of the time since death on the endogenous compound composition of human femoral blood samples. To this end, we have compiled a unique, exceptionally extensive postmortem data set consisting of 427 cases, each with paired blood samples (854 in total) collected at two different time points after death. This dataset should allow the systematic investigation of blood collection time after death and its relevance in future postmortem metabolome study designs.

## Experimental

### Chemical and reagents

Acetylcarnitine (C2), adenine, adenosine, alanine, arginine, carnitine (C0), cholic acid, cortisol, cortisone, creatinine, decanoylcarnitine (C10), dodecanoylcarnitine (lauroylcarnitine, C12), glycocholic acid, hexadecanoylcarnitine (palmitoylcarnitine, C16), hippuric acid, histidine, inosine, isoleucine, kynurenine, leucine, levothyroxine, lysine, methionine, octadecanoylcarnitine (stearoylcarnitine, C18), octanoylcarnitine (C8), ornithine, phenylalanine, proline, propionylcarnitine (C3), reserpine, riboflavin, serine, taurine, taurocholic acid, tetradecanoylcarnitine (myristoylcarnitine, C14), threonine, tryptophane, tyrosine, uracil, uric acid, valine, and 5,10,15,20-tetrakis-(pentafluorphenyl)-porphyrin were purchased from Sigma-Aldrich (Buchs, Switzerland). The lipids 1-palmiotyl-2-hydroxy-sn-glycero-3-phosphocholine (lyso PC 16:0), 1-oleoyl-2-hydroxy-sn-glycero-3-phosphocholine (lyso PC 18:1), 1-palmitoyl-2-oleoyl-sn-glycero-3-phosphocholine (PC 34:1), 1-stearoyl-2-linoleoyl-sn-glycero-3-phosphocholine (PC 36:2), 1-palmitoyl-2-oleoyl-sn-glycero-3-phosphoethanolamine (PE 34:1) and 1-palmitoyl-2-arachidonoyl-sn-glycero-3-phosphoethanolamin (PE 36:4) were purchased from Avanti Polar Lipids and were delivered by LuBio Science (Zurich, Switzerland). Deuterated and heavy-labeled internal standards (IS), arginine ^13^C6, creatinine N-methyl-D3, and phenylalanine-D1 (purity > 98%) were purchased from Cambridge isotope laboratories, which were delivered by ReseaChem Life Science (Burgdorf, Switzerland) or Sigma-Aldrich (Buchs, Switzerland). Water, acetonitrile (ACN) and methanol (MeOH) of HPLC grade were obtained from Fluka (Buchs, Switzerland). All other chemicals used were from Merck (Zug, Switzerland) and of the highest grade available.

### Postmortem femoral blood samples

Femoral blood samples from authentic forensic cases were collected at two time points after death during the routine toxicological investigation at the Victorian Institute of Forensic Medicine (VIFM), Melbourne, Australia. Upon mortuary admission of a deceased, approximately 2 to 5 mL of postmortem femoral blood was collected by leg puncture (blind stick) as soon as practicable, as per provisions of the Coroners Act 2008 (Victoria) (t1). A second femoral blood sample was collected during the medico-legal autopsy (t2) after preparation of the femoral vein. For cases where the time of death (ToD) could only be narrowed down to a specific day but no exact time (*n* = 163) and admission to the VIFM on a later day, ToD was defined as 12 pm of the estimated day of death. If admission to the VIFM was on the same day as the estimated day of death, ToD was specified to be between 12 am and mortuary admission of the body (t1). These timings were used to calculate the pre-admission and pre-autopsy intervals per case [defined as the time between death and sample collection at mortuary admission (t1) / autopsy (t2)]. All postmortem blood samples were preserved in 1% w/v sodium fluoride and potassium oxalate and stored at 4 °C until shipment. Samples were transported to the Zurich Institute of Forensic Medicine (ZIFM, Switzerland; exempt specimens, no import/export permission required) in a temperature-controlled environment at 4 °C and immediately frozen at − 80 °C upon receipt until re-analysis for drug and metabolome changes. Anonymized information on the estimated ToD and sampling time points were provided for further data analysis. From an initial 477 cases (Brockbals et al., 2021), 427 were included in the current study. Case selection was based on the detectability of one or more drugs (of abuse) independent to the cause of death. Re-analysis of the samples in an anonymized format for the specific research project was approved by the Ethics Committee of the VIFM (EC 20-2019; EC 23-1275). The individuals cannot be identified from the information provided. Hence, no written informed consent from the individuals or their relatives was needed for this study. Additionally, the study was conducted in full conformance with the Swiss ethical laws, particularly those covering the use of human material in research.

### Sample preparation

To 150 μL of postmortem blood, 15 μL IS solution (0.025 mM creatinine-d3, 0.03 mM L-arginine (13C6), and 0.04 mM L-phenylalanine-D1) were added, followed by the addition of 450 μL of a MeOH/acetone mixture (90:10 v/v) for protein precipitation. The samples were shaken and stored at − 20 °C overnight. Subsequently, the samples were resuspended, centrifuged at 14′000 rpm for 15 min, and one aliquot (50 μL) of the supernatant was transferred to an autosampler vial for analysis by reversed-phase chromatography (RP) as detailed below. A second aliquot (50 μL) was stored at − 80 °C for analysis by hydrophilic interaction chromatography (HILIC) approximately 1 month later. Before analysis, all samples were centrifuged again (14′000 rpm for 15 min).

In addition, a femoral blood pool sample was prepared from 11 authentic postmortem blood samples collected at the ZIFM, stored in aliquots at − 80 °C, thawed, and extracted identically to the study samples each day for quality control purposes.

### HR-MS analysis

Analysis was performed on a Thermo Fisher Ultimate 3000 UHPLC system (Thermo Fisher Scientific, San Jose, CA, USA) coupled with a high-resolution (HR) time of flight (TOF) instrument system (TripleTOF 6600 Sciex, Turbo V ion source, Concord, Ontario, Canada) as described in detail elsewhere (Boxler et al., 2019; Steuer et al., 2020).

Briefly, two chromatographic columns were applied, (a) a RP column (XSelect HSST RP-C18 column; 150 mm × 2.1 mm i.d; 2.5 µm particle size; Waters, Baden, Daettwil, Switzerland) with 10 mM ammonium formate and 0.1% (v/v) formic acid in water or 0.1% (v/v) formic acid in methanol as mobile phases A and B, respectively; gradient elution starting at 100% A with a flow rate of 0.5 mL/min, increase to 100% B between 1 and 15 min, held for 3 min and re-equilibrated for 2 min (0.7 mL/min flow rate after 15 min); 20 min total run time; (b) a Merck SeQuant ZIC HILIC column (150 mm × 2.1 mm i.d; 3.5 µm particle size) with 25 mM ammonium acetate and 0.1% (v/v) acetic acid in water and 0.1% (v/v) acetic acid in ACN as mobile phases C and D, respectively; gradient elution at a flow rate of 0.5 mL/min over 15 min; starting conditions were 95% D, decreased to 40% D between 1 and 10 min, further decreased to 10% D until 12 min, hold for 1 min and re-equilibrated for 4 min.

HR-MS (resolving power (full width at half-maximum, FWHM at 400 m/z) of 30,000) and MS/MS (resolving power 15,000 in MS^2^) data were acquired by data-dependent acquisition (DDA) after electrospray ionization (ESI) in positive mode for RP chromatography and negative mode for HILIC chromatography, respectively. The following settings were applied: full scan over a mass range from m/z 50 to m/z 1000 (accumulation time 50 ms, CE 5 eV) and MS2 scan (accumulation time for each DDA experiment 100 ms, CE 35 eV with a CE spread of 15 eV) after dynamic background subtraction on the five most intense ions with an intensity threshold above 100 cps and exclusion time of 5 s (half peak width) after two occurrences in high sensitivity mode. Data acquisition was controlled by Analyst TF software (version 1.7, Sciex).

All sample extracts were divided into 17 batches, with samples t1 and t2 from one case assigned to the same batch. Per batch, samples were measured in randomized order (total time period 1 month per chromatographic method). A system suitability test (SST) containing arginine, cortisol, cortisone, creatinine, glycocholic acid, hippuric acid, leucine, raffinose, riboflavin, and tryptophan (concentration 10 μg/ml each) was measured at the beginning of each measurement batch to check the general instrument performance via retention time and peak area comparison after peak integration in MultiQuant V 2.1 (Sciex). Automatic MS and MS/MS calibration was performed every 10 sample injections using a pooled blood sample (450 μL supernatant) fortified with 45 μL of a self-prepared calibration solution (creatinine, leucine, arginine, hippuric acid, tryptophane, inosine, cortisol, cortisone, riboflavin, glycocholic acid, taurocholic acid, reserpine, levothyroxine and 5,10,15,20-tetrakis-(pentafluorphenyl)-porphyrin, 7.1 μg/ml per analyte). Additionally, a pooled blood sample was repeatedly injected following each calibration and evaluated for intra- and inter-batch differences in retention time and peak area.

### Data processing and data analysis

Data analysis was done in a targeted approach through peak integration of 38 analytes (given in Table 1) in MultiQuant V 2.1 (Sciex). After raw data export to Microsoft Excel, further data analysis was performed using Microsoft Excel, GraphPad Prism 10.0.2, and R (R_Core_Team, 2023) in R Studio (R Version 4.3.1. “Beagle Scouts”; RStudio version 2023.03.0 + 386) with the following R packages: tidyverse (Wickham et al., 2019), gridExtra (Auguie, 2017), trelliscopeis (Hafen & Schloerke, 2021), flextable (Gohel & Skintzos, 2023), ggforce (Pedersen, 2022), readxl (Wickham & Bryan, 2023), and lubridate (Grolemund & Wickham, 2011).

**Table 1:** Overview of median percent changes of 38 tested endogenous compounds between paired blood samples (paired analysis, t2_t1) and between different time groups after death compared to group 1 (0–6 h)

Class	Analytes	Paired analysis		Unpaired analysis			RT RP	RT HILIC	Sum formula	Accurate mass	logP
		Median %change t2_t1		highest median %change	Time group highest %change						
Amino acid	Alanine	74	***	172	> 144 h	***	0.9	6.8	C3H7NO2	89.047678	− 2.85
Amino acid	Arginine	− 16	***	27	12–24 h	ns	0.8	9.4	C6H14N4O2	174.11167	− 4.2
Amino acid	Histidine	33	***	71	> 144 h	***	0.8	8.3	C6H9N3O2	155.06947	− 3.32
Amino acid	Leucine/ isoleucine	17	***	51	> 144 h	***	1.9	5.3	C6H13NO2	131.09462	− 1.52/ 1.70
Amino acid	Lysine	15	***	57	> 144 h	***	0.8	9.6	C6H14N2O2	146.10552	− 3.05
Amino acid	Methionine	14	***	45	> 144 h	***	1.3	5.6	C5H11NO2S	149.05104	− 1.87
Amino acid	Ornithine	− 12	***	− 25	72–96 h	***	0.7	9.7	C5H12N2O2	132.08987	− 4.22
Amino acid	Phenylalanine	17	***	59	> 144 h	***	3.9	5.1	C9H11NO2	165.07897	− 1.38
Amino acid	Proline	− 23	***	− 41	96–120 h	***	0.9	6.2	C5H9NO2	115.06332	− *2.7*
Amino acid	Serine	− 2	ns	23	> 144 h	***	0.9	7.1	C3H7NO3	105.04259	− 3.07
Amino acid	Threonine	0	ns	-15	24–36 h	***	0.9	6.8	C4H9NO3	119.05824	− 2.94
Amino acid	Tryptophane	9	***	42	> 144 h	***	5.0	5.4	C11H12N2O2	204.08987	− 1.06
Amino acid	Tyrosine	24	***	79	> 144 h	***	1.9	6.0	C9H11NO3	181.07389	− 2.26
Amino acid	Valine	− 8	***	− 14	24–36 h	***	0.9	5.9	C5H11NO2	117.07897	− 2.26
Carnitine	Carnitine (C0)	99	***	274	> 144 h	***	0.9	n.d	C7H15NO3	161.10519	− *2.9*
Carnitine	Decanoylcarnitine (C10)	− 63	***	-80	> 144 h	***	12.1	n.d	C17H33NO4	315.24095	− *0.85*
Carnitine	Lauroylcarnitine (C12)	− 37	***	− 54	120–144 h	**	13.3	n.d	C19H38NO4	344.27953	*0.03*
Carnitine	Myristoylcarnitine (C14)	8	*	73	72-96 h	**	14.2	n.d	C21H42NO4	372.31083	*0.86*
Carnitine	Palmitoylcarnitine (C16)	22	***	70	72-96 h	***	14.9	n.d	C23H46NO4	400.34213	*1.77*
Carnitine	Steraroylcarnitine (C18)	40	***	123	120–144 h	***	15.3	n.d	C25H50NO4	428.37343	*2.62*
Carnitine	Acetylcarnitine (C2)	82	***	227	72–96 h	***	1.1	n.d	C9H17NO4	203.11575	− *2.4*
Carnitine	Propionylcarnitine (C3)	42	***	98	72–96 h	***	2.2	n.d	C10H19NO4	217.13140	− *2.3*
Carnitine	Octanoylcarnitine (C8)	-11	**	35	72–96 h	ns	10.2	n.d	C15H29NO4	287.20965	− *1.2*
Amino acid	Creatinine	33	***	97	> 144 h	***	1.0	4.7	C4H7N3O	113.05891	− 1.76
Carbonyl compound	Kynurenine	3	**	62	96–120 h	***	3.7	n.e	C10H12N2O3	208.08479	− *1.9*
Organosulfonic acids	Taurine	141	***	379	120–144 h	***	0.9	6.7	C2H7NO3S	125.01466	− *2.2*
Bile acid	Cholic acid	166	***	1190	> 144 h	***	14.1	n.e	C24H40O5	408.28757	2.02
Nucleoside	Inosine	27	***	54	72–96 h	***	3.7	n.e	C10H12N4O5	268.08077	− 2.1
Pyrimidine	Uracil	74	***	176	> 144 h	***	1.5	2.1	C4H4N2O2	112.02727	− 1.07
Purine	Uric acid	8	***	49	> 144 h	***	1.7	6	C5H4N4O3	168.02834	− 2.17
Steroid	Cortisol	− 23	***	30	12–24 h	ns	11.1	n.e	C21H30O5	362.20932	1.61
Phospholipids	lyso PC 16:0	− 13	***	− 20	120–144 h	***	15.5	n.e	C24H50NO7P	495.33249	*1.83*
Phospholipids	lyso PC 18:1	− 21	***	− 38	120–144 h	***	15.6	n.e	C26H52NO7P	521.34814	*2.38*
Phospholipids	lyso PE 18:0	75	***	104	> 144 h	***	15.8	n.e			
Phospholipids	PC 34:1	− 1	ns	6	96–120 h	ns	18.0	n.e			
Phospholipids	PC 36:2	− 4	***	− 15	> 144 h	***	18.2	n.e			
Phospholipids	PE 34:1	29	***	53	> 144 h	***	17.6	n.e			
Phospholipids	PE 36:4	20	***	26	> 144 h	***	17.2	n.e			

Statistical testing was performed for paired testing by Wilcoxon signed rank test (*p* < 0.05) and by Kruskal–Wallis test (*p* < 0.05) for different time groups (unpaired). ns > 0.05, * < 0.05, ** < 0.01, *** < 0.001. logP values were taken from the Human Metabolome Database (HMDB), where italic values represent predicted rather than experimental values

*RP* reversed phase chromatography, *HILIC* hydrophilic liquid interaction chromatography, *n.d* not detectable, *n.e* not evaluated, logP values

#### Quality control

ISs were monitored to identify outliers (Grubbs test (GraphPad Prism 10.0.2) on batch-normalized IS peak areas, *p* < 0.05) and for quality control purposes, considering a variation (relative standard deviation, RSD) of < 30% as sufficiently robust among the authentic samples. The mean and range of retention times and peak areas of all 38 analytes were determined in the pool samples. Intra- and inter-batch differences were calculated using Microsoft Excel. Deviations (standard deviation) of a maximum of 0.05 min or 0.2 min, and 20% or 30% in peak areas were considered acceptable within and between batches, respectively.

#### Evaluation of normalization procedures

To account for inter-batch differences originating from technical variation, all analyte peak areas were normalized to the mean (*n* = 5) of the batch’s pool-sample analyte peak area (batch correction). Two different sample normalization strategies were evaluated: normalization to heavy-labeled ISs (IS-normalization) and probabilistic quotient normalization (PQN).IS-normalization was performed by dividing the analyte’s peak through the IS area. Metaboanalyst 6.0 (Pang et al., 2024) was used for PQN normalization of the whole data set (38 analytes, 854 samples).

#### Postmortem changes between two time points of the same case (paired)

Percent differences of raw peak areas between t2 and t1 were calculated for each case (*n* = 427) and analyte. Subgroups, in terms of increasing time intervals, were formed according to the time difference (Δ*t*) between t2 and t1 as follows: 0–12 h,12–24 h, 24–36 h, 36–48 h, 48–72 h, 72–96 h, 96–120 h, 120–144 h, and > 144 h. A paired Wilcoxon signed ranked test (*p* < 0.05; ns > 0.05, * < 0.05, ** < 0.01, *** < 0.001; p-values adjusted for multiple testing according to “holm”) was applied between t2 and t1 peak areas for all cases and in Δ*t* subgroups.

#### Postmortem changes over time (unpaired analysis)

Subgroups were formed according to the time difference of each individual blood sample (tx_ToD, *n* = 854) to the known or estimated ToD as follows: 0–6 h (group 1), 6–12 h (group 2), 12–24 h (group 3), 24–36 h (group 4), 36–48 h (group 5), 48–72 h (group 6), 72–96 h (group 7), 96–120 h (group 8), 120–144 h (group 9), > 144 h (group 10). Statistical differences between groups were assessed by application of a Kruskal Wallis test (*p* < 0.05; ns > 0.05, * < 0.05, ** < 0.01, *** < 0.001) followed by Dunn’s multiple comparison test (*p* < 0.05; ns > 0.05, * < 0.05, ** < 0.01, *** < 0.001) after false-discovery rate correction by the Holm method. Percent differences of the median normalized peak area of each group to group 1 (0–6 h) were calculated.

#### Correlations

The percent changes (paired and unpaired analysis) were correlated with the possible influencing factors logP (lipophilicity), molecular weight (MW), and retention time in two different chromatographic settings by Spearman correlation analysis in GraphPad Prism 10.0.2. The corresponding characteristics and references used for correlations are summarized in Table 1.

## Results

### Sample cohort and analysis

The sample cohort consisted of blood samples from 427 authentic forensic cases with two collection time points after death (t1 and t2) per case (*n* = 854 blood samples). Median (and range) collection times after death were 8 h (1.3–290 h) for t1, and 88 h (11–478 h) for t2, respectively, resulting in a median Δt between t1 and t2 samples of 71 h (6.4–434 h). The different sampling procedures for t1 and t2 revealed no statistically significant differences for 24 analytes when exemplarily comparing t1 and t2 samples collected between 24 and 36 h (best-balanced time-group with *n* = 33 t1 vs. *n* = 45 t2 samples, nonparametric Mann–Whitney test, *p* < 0.05). The 12 analytes with significant findings, all pointed towards lower concentrations at t2 (median difference − 31%). Manner of death was natural in 195 cases, accidental in 69 cases, suicide in 57 cases, and remained unknown in 106 cases. The age of the deceased at the ToD ranged from 15 to 98 years (mean/median 59 years). No correlation could be observed between age of the deceased and the time between death and t1 (Spearman rank correlation coefficient: 0.23, linear model R^2^ = 0.04; data not shown). Additionally, no trend was found that would indicate longer/shorter time intervals until first sample collection or Δt with different manner of deaths (data not shown in detail). All cases included in the current study tested positive for at least one drug or alcohol during a comprehensive routine drug screening (Di Rago et al., 2021); 210 for opioids, 216 for benzodiazepines, 216 for antidepressants, 97 for antipsychotics, 43 for cannabis, and 36 for stimulants (amphetamines, cocaine). Significant influences of storage and shipping conditions were considered negligible, as shown in a preceding study (Brockbals et al., 2021). QTOF analysis allowed for sufficient targeted processing of 38 endogenous compounds following separation by standard RP chromatography. For analytes with a low RP retention time, trends in time-dependent changes were confirmed by HILIC chromatography mode before inclusion in the results (*n* = 18 analytes as detailed in Table S2).

### Quality control and evaluation of normalization strategies

IS were used for quality assessment throughout the analytical batch. A performed Grubbs test indicated three samples as potential outliers based on one out of the three IS, but no sample was classified as a potential outlier for all three IS. IS RSDs as well as RSDs of all analytes within the QC pools and the authentic samples are provided in the supplementary information (Table S1). The set criteria of ± 30% for pool samples and IS were fulfilled for all analytes in RP mode except for (lyso)phospholipids, serine, and threonine.

**Fig. 1:**
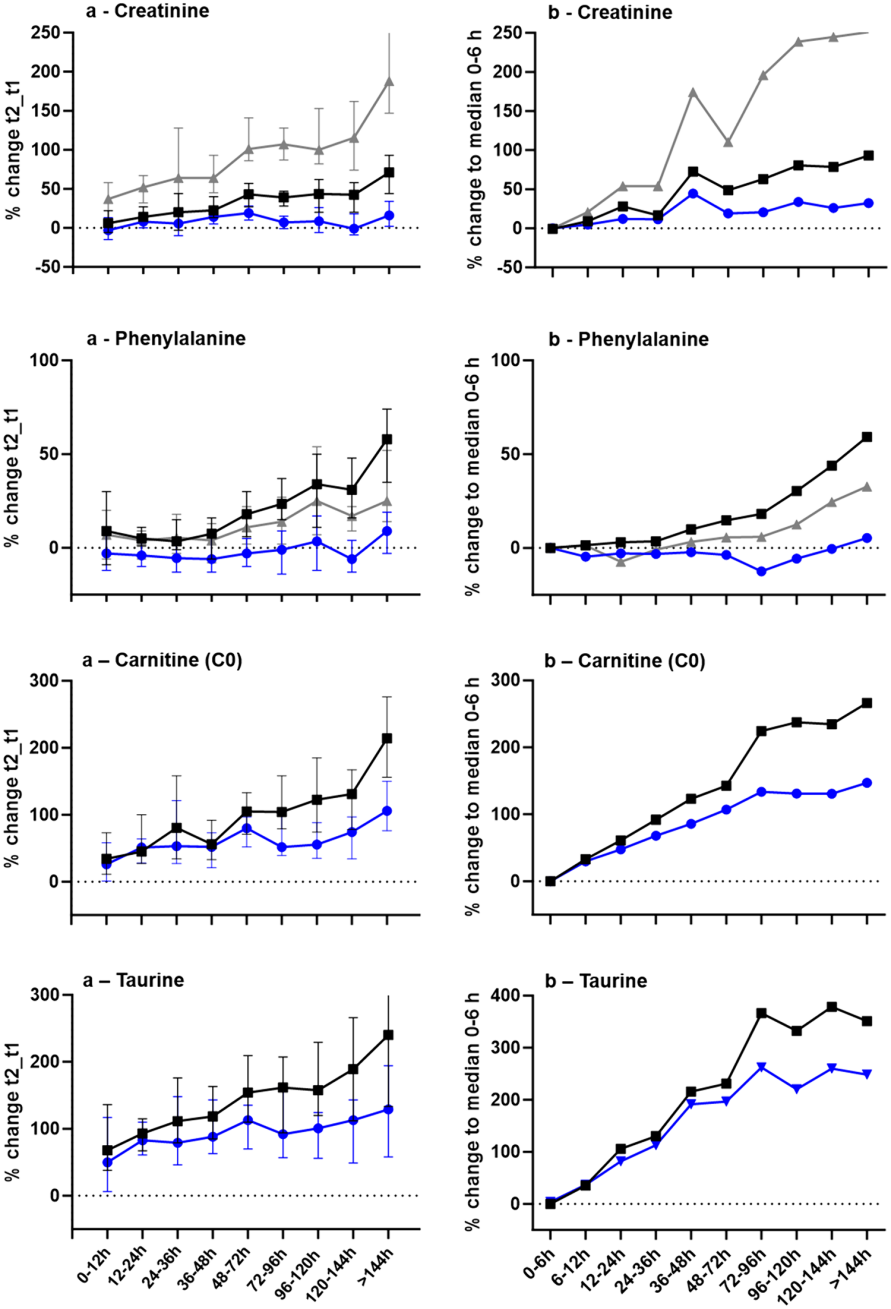
Evaluation of different data normalization strategies: only batch-correction (black squares), PQN (blue circles), and IS-correction (grey triangles) for paired analysis between t2 and t1 (**a**) and unpaired analysis of individual blood samples (**b**). For (**a**), median and range (95% confidence interval) of percent change between t2 and t1 according to the different time interval lengths (12 or 24 h intervals) are depicted. For (**b**), median percent changes of the corresponding time group to group 1 (0–6 h) are given. The dotted line represents no change; values above zero increase with more extended time intervals, and values below zero decrease, respectively. GraphPad Prism 10.0.2 was used for figure creation

All samples were batch-corrected to account for instrument variation over a 1-month measuring period. In addition, two common normalization strategies were evaluated. In targeted (semi-quantitative) analysis, using the respective isotopically labelled IS of an analyte represents the gold standard to account for variation resulting from the laboratory handling, while PQN is a common normalization method in untargeted analysis accounting for many features (Dieterle et al., 2006). Effects of the different procedures are exemplified in Fig. 1 for two compounds with matching heavy-labelled IS (creatinine, phenylalanine), and two additional compounds with high time-dependent effects in the current study (C0 and taurine), for paired (A) and unpaired analysis (B). General trends of increasing concentrations over time and observed significant differences remain comparable among the two normalization approaches compared to only batch-corrected data, while the magnitude of change is lower for PQN normalization.

### Postmortem changes in endogenous compounds between two time points of the same case (paired; t2 vs. t1)

**Fig. 2:**
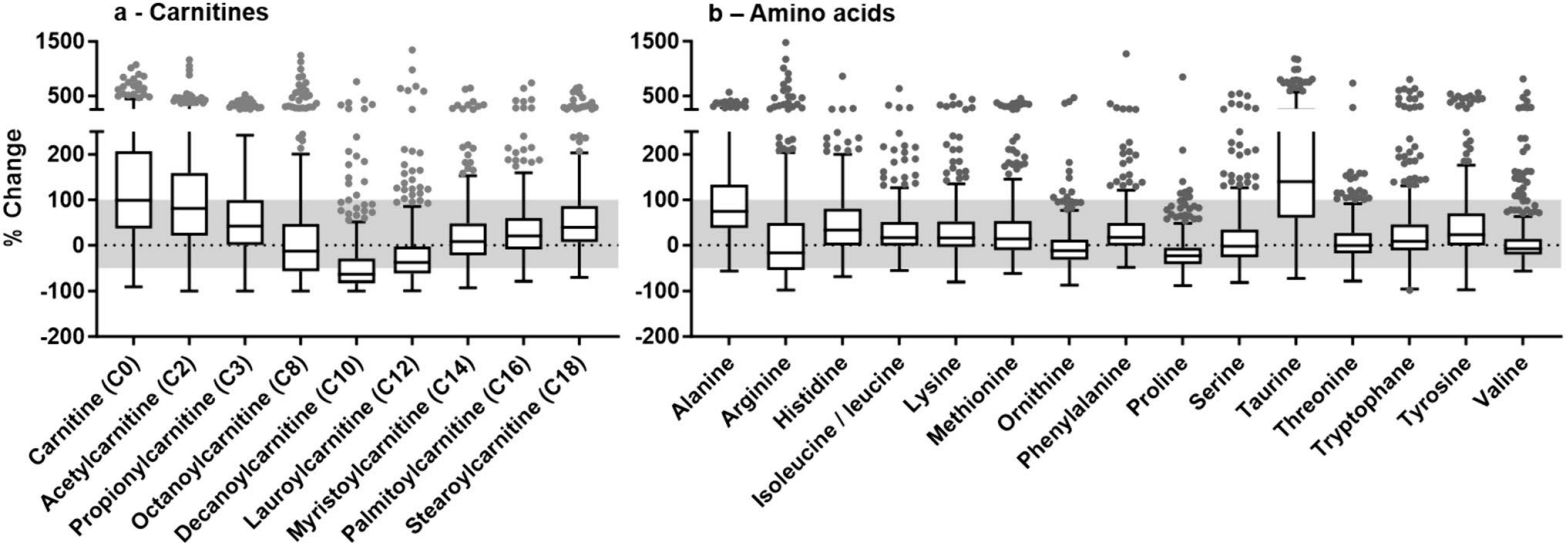
Boxplots of percent differences between t2 and t1 in the individual cases (paired samples) for acylcarnitines (**a**) and different amino acids (**b**). Zero (dotted line) represents no change between the two time points, while the grey area corresponds to differences between − 50% and + 100% (fold-change of two). GraphPad Prism 10.0.2 was used for figure creation

An overview of medianpercent differences for the chosen 38 analytes over all analyzed cases is provided in Table 1. Exemplified for (acyl)carnitines with increasing carbon side chain length and different amino acids, the extent and distribution of time-dependent changes is depicted in Fig. 2; for all other analytes visual representation can be found in the Supplementary information in Fig. S1. Except for serine, threonine, and PC 34:1, all compounds revealed significant differences between t2 and t1 (*p* < 0.05). For octanoylcarnitine (C8), decanoylcarnitine (C10), lauroylcarnitine (C12), arginine, ornithine, proline, valine, cortisol, lyso PC 16:0, and lyso PC 18:1 median decreases were observed, while all other analytes showed significant median increases from t1 to t2. Overall, changes mainly ranged from− 50% to + 100% (corresponding to a fold change of two) when considering their interquartile range. Exceptions presented carnitine (C0), acetylcarnitine (C2), decanoylcarnitine (C10), lauroylcarnitine (C12), alanine, taurine, cholic acid, uracil, and lyso PE 18:0. Here, maximum median differences between t2 and t1 reached from − 63% [decanoylcarnitine (C10)] to 166% (cholic acid) and 141% (taurine). Still, large inter-individual variations were observed for all samples and also subgroups with increasing Δt intervals (< 6 h—> 144 h) (see supplementary information Table S2).

Endogenous compounds were categorized into four patterns of median changes depending on the length of the Δt:

- Steady increase: alanine, creatinine, proline, tryptophane, taurine, uracil, valine, carnitine (C0), acetylcarnitine (C2), propionylcarnitine (C3), steraroylcarnitine (C18), lysoPE 18:0, PE 34:1, PE 36:2.
- Constant median change for the time intervals of approximately 24 to 36 h followed by an increase with longer Δt: histidine, leucine/isoleucine, lysine, methionine, phenylalanine, tyrosine, uric acid, palmitoylcarnitine (C16), cholic acid.
- Decrease: cortisol, decanoylcarnitine (C10), lauroylcarnitine (C12).
- No or < 30% change over time: arginine, inosine, ornithine, serine, threonine, octanoylcarnitine (C8), myristoylcarnitine (C14), kynurenine, lysoPC 16:0, lysoPC 18:1, PC 34:1, PC 36:2.

**Fig. 3:**
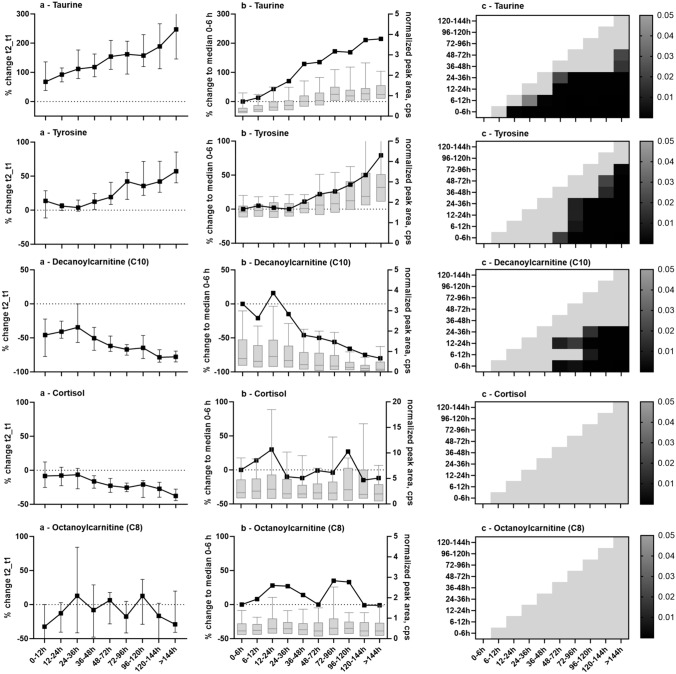
**a** Median and range (95% confidence interval) of percent change between t2 and t1 according to the different time interval lengths (12 or 24 h intervals). The dotted line represents no change; values above zero increase with more extended time intervals, and values below zero decrease, respectively. **b** Median percent change of the corresponding time group to group 1 (0–6 h, black squares). Light grey boxplots indicate normalized peak areas per time group. The dotted line represents no change; values above zero increase with more extended time intervals, and values below zero decrease, respectively. **c** *p*-value heat map highlighting significant changes determined by Dunn’s multiple comparison post-hoc test (*p* < 0.05) between individual postmortem time groups. Light grey area indicates non-significant results, dark grey to black areas indicate significant changes (*p* < 0.05), with darker areas representing a lower p-value. GraphPad Prism 10.0.2 was used for figure creation

Representative examples are depicted in Fig. 3a for taurine taurine (a), tyrosine (b), decanoylcarnitine (C10) and cortisol (c), and octanoylcarnitine (C8) (d).

### Postmortem changes in endogenous compounds according to their time since death (unpaired; tx_ToD)

To determine whether the actual time after death plays a decisive role, or is even more important than the time interval between t2 and t1, all samples (*n* = 854) were binned into groups according to the individual samples’ time since death (ToD to t1 and ToD to t2, tx_ToD) and were statistically compared for differences in an analyte’s normalized peak area. In nine cases, t1 and t2 blood samples were binned within the same group therefrom five cases had a sampling time > 144 h. A Kruskal–Wallis test revealed significant changes between above mentioned groups for all tested endogenous compounds except for arginine, octanoylcarnitine (C8), cortisol, and PC 34:1 (Table 1). Median percent differences of each group (1–10) to group 1 (0–6 h, earliest) are summarized in Table S2 of the Supplementary information. The highest median changes were observed for carnitine (C0) (+ 274%), taurine (+ 361%), and cholic acid (+ 1190%), as well as decanoylcarnitine (C10) (− 80%) and lauroylcarnitine (C12) (− 42%).

**Fig. 4:**
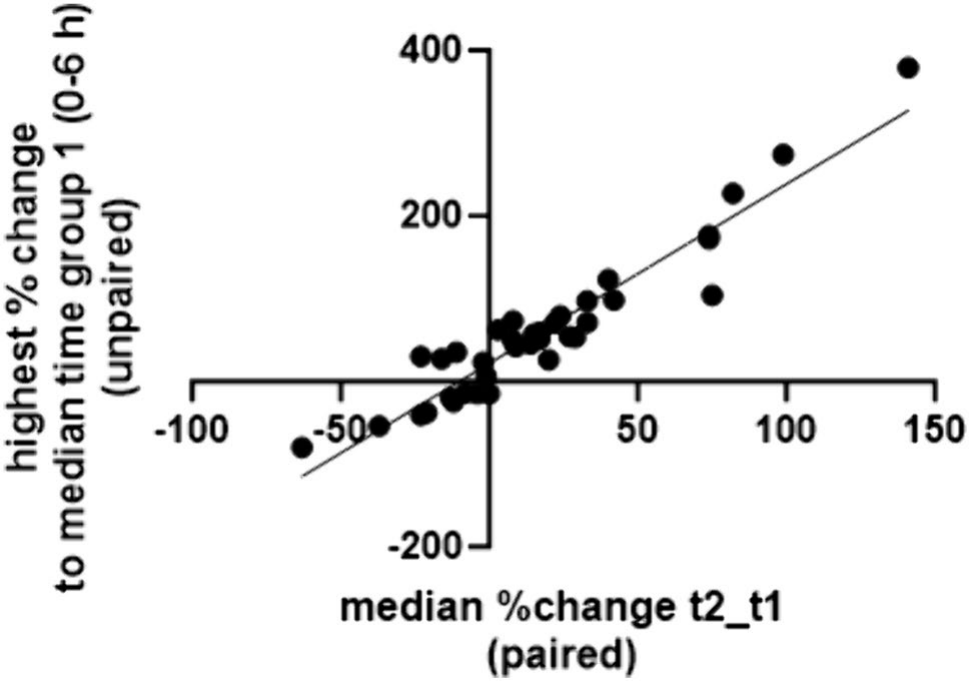
Correlation between the median percent change between t2 and t1 (paired analysis, x-axis) and the highest percent change compared to time group 1 (0–6 h, unpaired analysis, y-axis) for the 38 endogenous compounds. Correlation analysis was done by Spearman correlation, R^2^ = 0.91). Each data point represents one endogenous compound. GraphPad Prism 10.0.2 was used for figure creation

As shown in Fig. 4, the correlation per analyte of its median Δt changes from paired analysis to the highest median %change of each group (2–10) to group 1 (unpaired analysis) indicated good agreement (spearman correlation R^2^ 0.91) despite other expected influencing factors in unpaired analysis. Figure 3b exemplifies box plots of the normalized peak area (left y-axis) and the median percentage change to time group1 (right y-axis). Boxplots of all other analytes are presented in Fig.S2. Also, for individual compounds, the time-dependent changes of the unpaired samples matched well with paired Δt data (Fig. 3a,b). The exception was cortisol, which decreases significantly between t2 and t1 (paired analysis) but showed no trend in the normalized peak areas with respect to the respective time since death of the blood samples. In contrast, myristoylcarnitine (C14), and lyso PC18:1 showed no trend as a function of Δt length but an increase or decrease as a function of time since death (Table S2).

For statistical analysis between all groups, Dunn’s post-hoc test was applied on Kruskal–Wallis significant analytes. Significant differences are given as a so-called *p*-value heat map for the chosen examples in Fig. 3c and all remaining compounds in the Supplementary information in Fig. S3. Significant changes between groups most often appeared with increasing time since death, while the initial 36 or even 48 h indicated relatively stable normalized peak areas. Few exceptions were observed for alanine, taurine, tryptophan, valine, carnitine (C0), and acetylcarnitine (C2), in line with findings from paired analysis between t2 and t1.

### Correlations

**Fig. 5:**
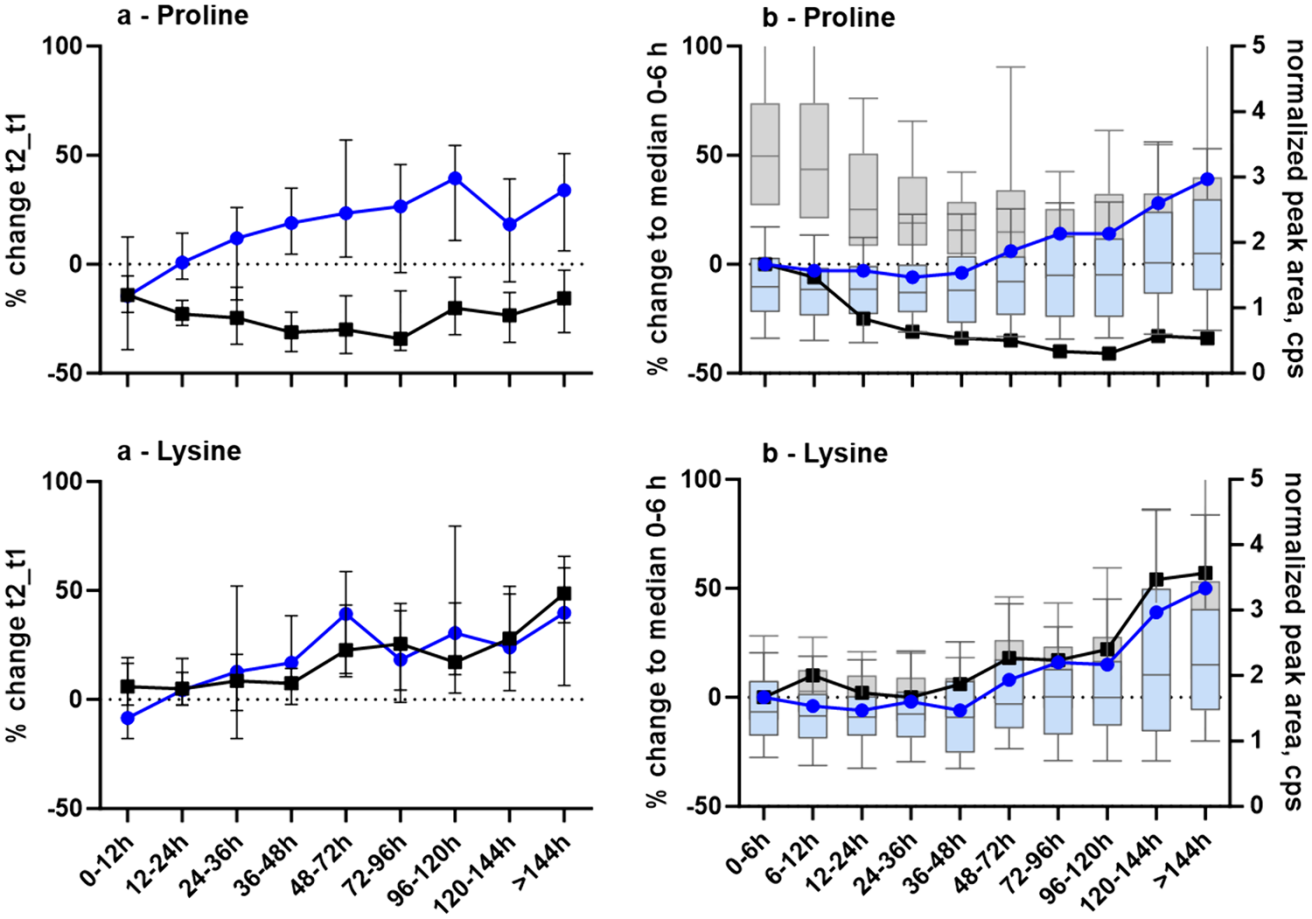
**a** Median and range (95% confidence interval) of percent change between t2 and t1 according to the different time interval lengths (12 or 24 h intervals). Results from RP chromatography are given in black squares, those of HILIC chromatography in blue circles. **b** Median percent change of the corresponding time group to group 1 (0–6 h, black squares for RP, blue circles for HILIC chromatography). Light grey boxplots indicate normalized peak areas per time group in RP chromatography, light blue boxplots for HILIC chromatography. The dotted line represents no change; values above zero increase with more extended time intervals, and values below zero decrease, respectively. GraphPad Prism 10.0.2 was used for figure creation

To find the underlying causes for the varying behavior between different compounds, the percent changes (paired and unpaired analysis) were correlated with the possible influencing factors. No correlations existed between percent change and lipophilicity or molecular weight (supplementary information Fig. S4A, B). In RP chromatography, the highest percent changes occurred for compounds eluting in the first two minutes and around 15 min of the chromatogram (Fig. S4C). HILIC chromatography (ESI negative) used for selected compounds indicated a similar extent of percental change despite compound elution around five to ten minutes. Further direct comparison of the observed postmortem changes for 18 compounds between RP and HILIC chromatography (Tables S2 and S3) also did not find any differences caused by the applied chromatography, with the exception of proline and valine. Both amino acids revealed increases in HILIC chromatography, in line with other amino acids, and appeared to be stable or slightly decreased when analyzed in RP mode. Examples from lysine (no difference, retention time RP 0.8, HILIC 9.6, respectively) and proline (different postmortem behavior, retention time RP 0.9 min, HILIC 6.2 min, respectively) are given in Fig. 5.

## Discussion

(Un)targeted metabolome approaches have gained significant interest in forensic toxicology analysis, including postmortem cases (Bonicelli et al., 2022; Brockbals et al., 2020, 2021; Chighine et al., 2023; Donaldson & Lamont, 2013, 2014; Elmsjo et al., 2021, 2022; Locci et al., 2019, 2021; Mora-Ortiz et al., 2019; Pesko et al., 2020; Peyron et al., 2019). Due to study design and ethical restrictions in controlled human studies, postmortem research typically involves random routine cases. However, the metabolome is highly dynamic and, even in living people, susceptible to many environmental factors influencing the metabolic profile or particular biomarkers. Postmortem specimens such as blood represent an even greater challenge given the well-recognized issues of postmortem changes or PMR, seen with drugs (Butzbach, 2010; Drummer & Gerostamoulos, 2023; Mantinieks et al., 2021; McIntyre & Escott, 2012; Pelissier-Alicot et al., 2003; Peters & Steuer, 2018; Skopp, 2010). So far, little is known about such (additional) confounding factors originating from death itself, but severe influences are expected, particularly from the time since death. A better understanding of these factors will significantly improve the experimental design of future postmortem metabolome studies.

Our current study comprised one of the most extensive data sets in the context of postmortem studies and is characterized by two blood collection time points per case. Despite the non-controlled sample collection, the study cohort can be considered representative of typical forensic postmortem cases, as different manners of death, a large age range, and a wide variety of collection time points were included. No systematic differences or correlations in age of the deceased or manner of death in relation to the PMI were found.

Thirty-eight endogenous compounds were chosen for detailed, time-dependent evaluation of postmortem changes from an untargeted acquired data set. These included metabolites of different compound classes with significantly different physicochemical properties, such as amino acids, acylcarnitines, (lyso)phopsholipids, bile acids, steroids, etc. The targeted processing method originally (during method development) included more endogenous compounds, from which we focused on analytes that could be measured with sufficient analytical precision. Of those, some could not reliably be detected in the postmortem sample cohort and were dropped subsequently, e.g. the nucleobase adenine and the nucleoside adenosine (Boxler et al., 2019). Of course, it is only a small selection of analytes and not representative of the complete metabolome. However, the chosen targeted compounds were previously described in (postmortem) or generally forensic metabolome studies. They were proposed as predictive biomarkers, e.g., as intoxication markers for oxycodone poisoning (Elmsjo et al., 2022) or the postmortem interval (Donaldson & Lamont, 2014; Mora-Ortiz et al., 2019). Batch normalization was performed based on pooled sample peak areas measured within the same batch to account for analytical bias. In addition, individual sample normalization to account for, e.g., extraction effects is commonly applied. For targeted (semi-/quantitative) analysis, matching isotopically labelled IS per analyte represent the gold standard for normalization. In (targeted) metabolomics, most often, such IS are not available for all compounds of interest. If a general IS (isotopically labelled, but not matching the analyte of interest) is used to normalize another analyte, the analyte of interest needs careful evaluation during method development and validation, and in the worst case the use of a general IS can increase variation rather than compensate for it (Boxler et al., 2019). In untargeted metabolomics, where compounds of interest are a priori unknown, specific or general IS-use is therefore unfeasible. PQN was demonstrated as a versatile sample normalization strategy of untargeted datasets of thousands of features, where a quotient for each feature is calculated in relation to a reference sample (pool), and the median of all feature quotients is used as a sample’s individual normalization/dilution factor (Dieterle et al., 2006). However, PQN can be biased, if, e.g., a large proportion of the features are changed because of a systematic rather than a dilution/extraction variation effect (Correia et al., 2022). In this current semi-targeted analysis, only 38 compounds were evaluated, from which several were already described to show PMI-dependent changes (Donaldson & Lamont, 2014; Mora-Ortiz et al., 2019). It is therefore possible, that PQN might attribute actual effects of the PMI to dilution effects, consequently underestimating the real time- dependent effect (Fig. 1). Given the descriptive nature of the current study, the final data evaluation was based on batch-normalized data only, to avoid overfitting effects of PQN normalization.

When performing a paired analysis of two blood samples from the same individual, it could be proposed that variables other than the time could be excluded. All case-specific parameters, like cause or manner of death, age, etc., remain identical. However, looking only at the %Δt change between t2 and t1 left out the actual time effect, i.e., the time since the death occurred. For instance, specific forensic postmortem cases can have a time difference between t1 and t2 of 12 h, but information, if death occurred one, two, or more days before blood collection, is not considered. We, therefore, additionally compared metabolite changes according to the time since death, although higher variability can be expected. Individual analysis increased the total number of samples to 854, as t1 and t2 were evaluated separately. Both data evaluation strategies (time intervals between paired t1 and t2 samples vs. unpaired analysis in groups according to the time since death of individual samples) returned very well-matching results (Figs. 3, 4). Cortisol poses one crucial example, where paired data evaluation was able to indicate time-dependent changes, but evaluation of random (non-paired) samples showed no trend. Generally, increases outweighed decreases over time (Table 1). Concerning the median and interquartile changes, almost all analytes ranged between a fold change of plus/minus two, but inter-individual variation was high (Figs. 2, and S1). Taurine and uracil, as two compounds exceeding the described range and showing time-dependent concentration increases, were already described as potential biomarkers of the PMI. However, in contrast to the current results, concentration decreases over time were described in mice (Mora-Ortiz et al., 2019).

If univariate statistics are employed for metabolome data evaluation in controlled studies of living people, often fold-changes of 1.5 or 2 are used as one of several filter criteria for interesting features. Considering our (paired) results, it was shown that already the time factor can introduce such variation for some analytes (Fig. 2). Depending on the research question and sample selection, higher fold-changes might be advisable in postmortem metabolome analysis to improve biological significance and avoid random findings. As in line with former works (Chighine et al., 2021), PMI is one of the main influencing factors on the metabolome; controlling or accounting for different PMIs within the study cohort is highly important for future postmortem metabolome studies. Our data suggest that the influence of PMI is most homogenous within the first 48 h after death. As such, the most reliable results would be obtained if a sufficiently high number of blood samples taken within the first 48 h after death can be used, ensuring the least influence of the PMI on concentration changes of endogenous analytes. Alternatively, PMI among study groups should be as balanced as possible.

Using correlation analysis, we attempted to find causes for the observed differences in postmortem behavior depending on the substance. Based on existing knowledge of exogenous compounds, e.g., lipophilicity, the volume of distribution (Vd), or the ratio of cardiac to peripheral blood (C/P-ratio) can help predict PMR (Han et al., 2012; Skopp, 2010), we aimed to compare different chemical properties of the endogenous compounds. Thereby, Vd is not available for endogenous metabolites, as they are typically not administered in known amounts to calculate their expected blood/plasma concentration in relation to the dose. C/P ratios or general distribution of endogenous metabolites would be interesting for further investigation of underlying PMR mechanisms but was out of scope for the current study that focused on femoral blood samples only. No correlations between logP or molecular weight and the extent of postmortem change could be observed (Fig. S4). Comparison of retention time and %change pointed towards more severe postmortem changes for those analytes eluting within the first two minutes of the RP chromatography. This could be due to similar physicochemical properties of these substances but also due to matrix effects. Typically, the first three minutes, as well as the end of a RP chromatography, are prone to matrix effects, given salts and extremely polar vs. highly lipophilic compounds (phospholipids), respectively (Van Eeckhaut et al., 2009).

Further, it is well known that postmortem samples are more susceptible to matrix effects than samples of living persons (Drummer, 2007; Saar et al., 2009). Eighteen analytes were additionally evaluated in a different chromatographic system (HILIC) and ESI negative ionization, a typically complementary method, to exclude matrix effects as the leading cause of the observed time-dependent changes. Only for proline and valine, a different time-dependent behavior was observed when changing the analytical methodology, which points towards a matrix effect for these two compounds in RP chromatography (Fig. 5, Tables S2, S3). Apart from that, HILIC has led to the same results as RP (Figs. S4 and  5, Tables S2, S3), but with overall higher variation. So far, no common physico-chemical properties could be deduced, allowing for a likelihood prediction of postmortem changes in endogenous metabolites.

The water content of postmortem blood samples demonstrates high variation ranging from 60 to 90% (Skopp, 2004), possibly contributing to a certain (minor) extent to the observed concentration changes. PQN normalization could compensate for these effects, however, as discussed above, more compounds/features in an untargeted data processing workflow will be necessary for a conclusion.

The changes in endogenous substances probably occur due to the lack of energy after death, accompanied by the cessation of aerobic and partial continuation of anaerobic metabolic pathways. Other studies in animal models found modulations in metabolites associated with anaerobic metabolism, such as lactate (Mora-Ortiz et al., 2019). In the broadest sense, our results also confirm previous studies with limited numbers of animals showing significant increases in amino acid levels. Different underlying mechanisms were discussed, among others, protein catabolism in postmortem cells leading to accumulation in blood after cell lysis or decreased protein synthesis (Donaldson & Lamont, 2014). A higher number of endogenous compounds or an untargeted data evaluation will be necessary to uncover biological mechanisms, e.g., through pathway analysis. Ideally, future research should apply an adapted experimental setup to include/extend the analysis to macromolecules (carbohydrates, proteins, lipids, DNA or RNA) and different (blood-surrounding) tissues that might influence concentrations of small endogenous molecules through distribution, changes in protein binding, or general postmortem degradation.

## Conclusions

The current study comprises one of the most extensive data sets in the context of time-dependent postmortem studies focusing on endogenous compounds. Comparable to drugs, we observed changes in blood levels of nearly all endogenous compounds in a time-dependent manner after death. Our paired analysis of two individual blood samples collected from the same individual proved highly valuable, as time since death represents the only variable. Additional unpaired sample evaluation purely based on the time since death, generally indicated, similar results to those of the matched time intervals, despite more confounders and higher variation. As PMI is one of the main influencing factors on postmortem metabolome changes, controlling or accounting for different PMIs within the study cohort is highly important for future postmortem metabolome studies. Most reliable results can be expected if blood samples preserved within the first 48 h after death can be used and/or PMI among study groups is balanced.

### Supplementary Information

Below is the link to the electronic supplementary material.

Supplementary file1 (PDF 2767 KB)

## Data Availability

As routine samples from forensic investigations were analyzed, data acquired during this study is not intended to be publicly available but can be provided upon reasonable request to the corresponding author.
