# Supplementary material for: Postmortem metabolomics: influence of time since death on the level of endogenous compounds in human femoral blood. Necessary to be considered in metabolome study planning?
Source: Metabolomics. 2024 May 9;20(3):51. doi: 10.1007/s11306-024-02117-y (PMC11081988; doi:10.1007/s11306-024-02117-y)
Supplement: Supplementary file 1 — Supplementary file1 (PDF 2767 KB) [file 11306_2024_2117_MOESM1_ESM.pdf]

Electronic supplementary information

Postmortem Metabolomics: Influence of time since death on the level of endogenous compounds in human femoral blood. Necessary to be considered in metabolome study planning?

Andrea E. Steuer[1*], Yannick Wartmann[1], Rena Schellenberg[1], Dylan Mantinieks[3,4], Linda L. Glowacki[4], Dimitri Gerostamoulos[3,4], Thomas Kraemer[1], and Lana Brockbals[1,2]

[1] Department of Forensic Pharmacology and Toxicology, Zurich Institute of Forensic Medicine, University of Zurich, Switzerland

[2] Centre for Forensic Science, School of Mathematical and Physical Sciences, Faculty of Science, University of Technology Sydney, Australia

[3] Department of Forensic Medicine, Monash University, Victoria, Australia

[4] Victorian Institute of Forensic Medicine, Victoria, Australia

***Correspondence**

Andrea Steuer

Department of Forensic Pharmacology and Toxicology, Institute of Forensic Medicine,

University of Zurich, Winterthurerstrasse 190/52, 8057 Zurich, Switzerland

+41 446355679

andrea.steuer@irm.uzh.ch

Content of Supplementary Information

Fig.S1 **page 2**

Fig. S2 **page 3**

Fig. S3 **page 9**

Table S1 **page 10**

Table S2 **page 11**

Table S3 **page 24**

Fig. S1: Boxplots of percent differences between t2 and t1 in the individual cases for different compounds. Zero (dotted line) represents no change between the two time points, while the grey area corresponds to differences between -50% to +100% (fold-change of 2). GraphPad Prism 10.0.2 was used for figure creation.

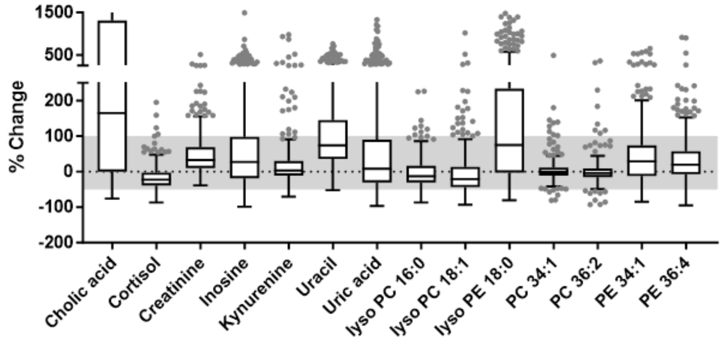

Fig S2: Box plots of normalized peak areas of individual blood samples according to different blood collection time groups after death. Figures were created in R.

a: amino acids

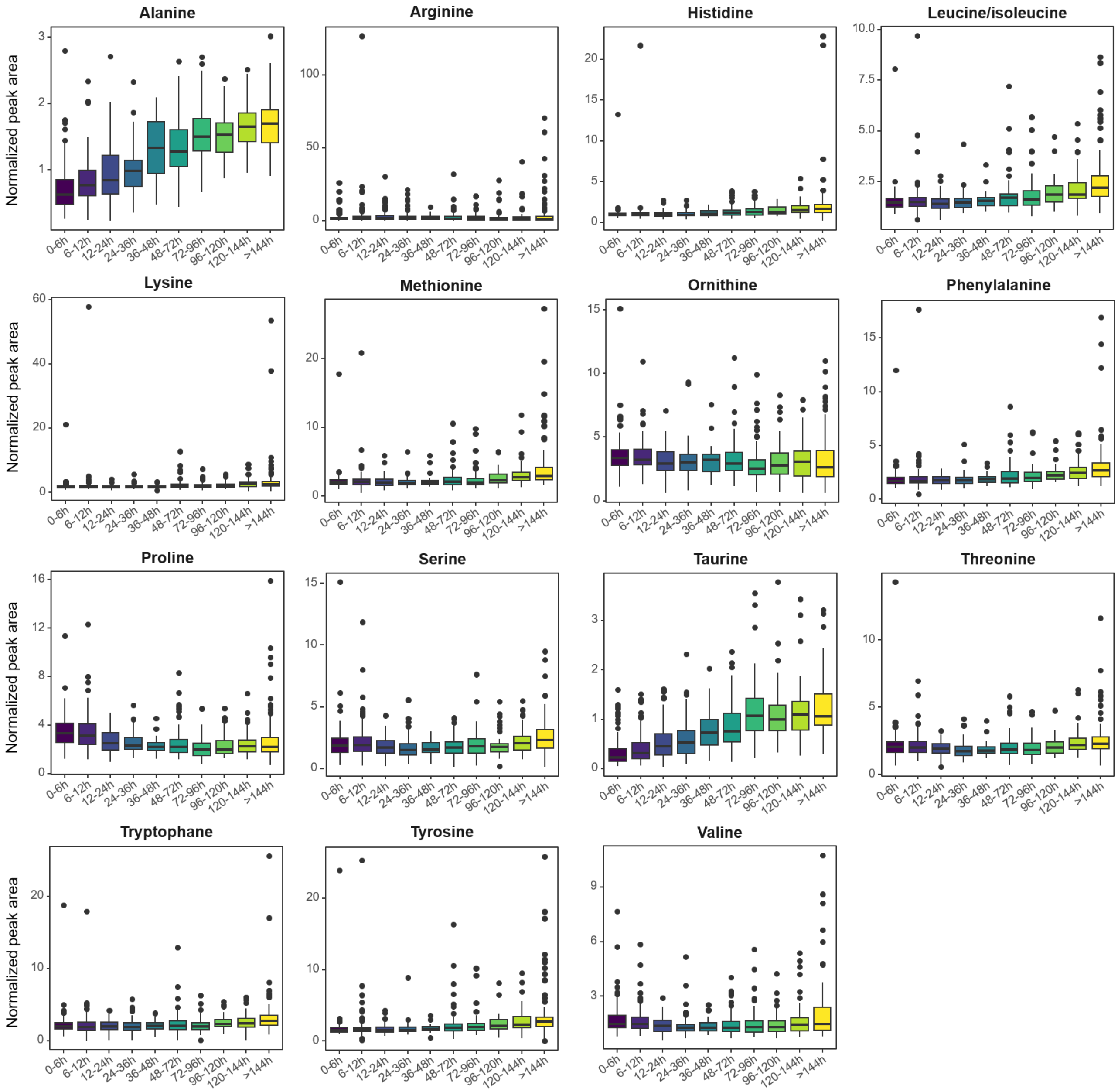

b: carnitines

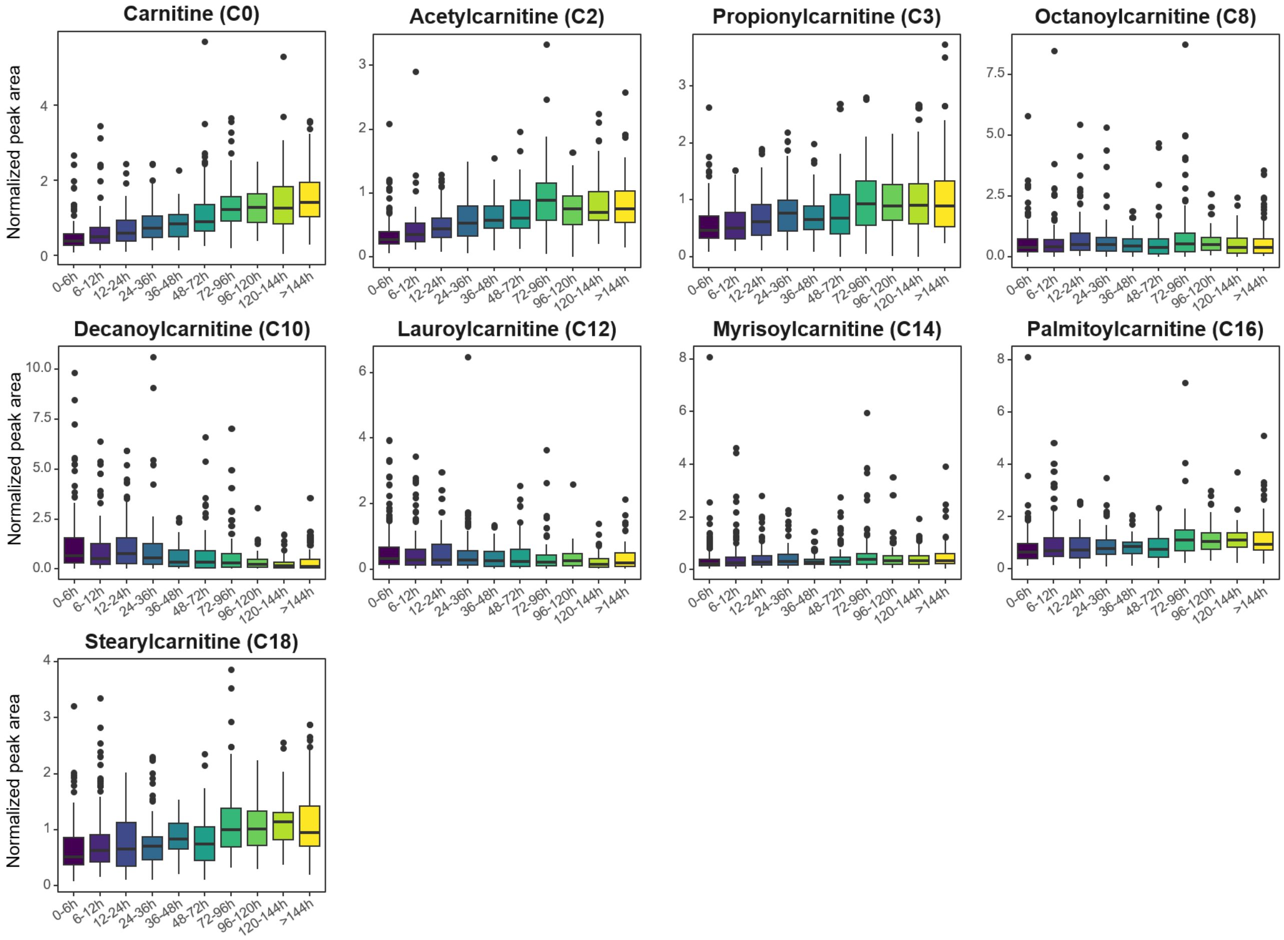

c: other compound (classes)

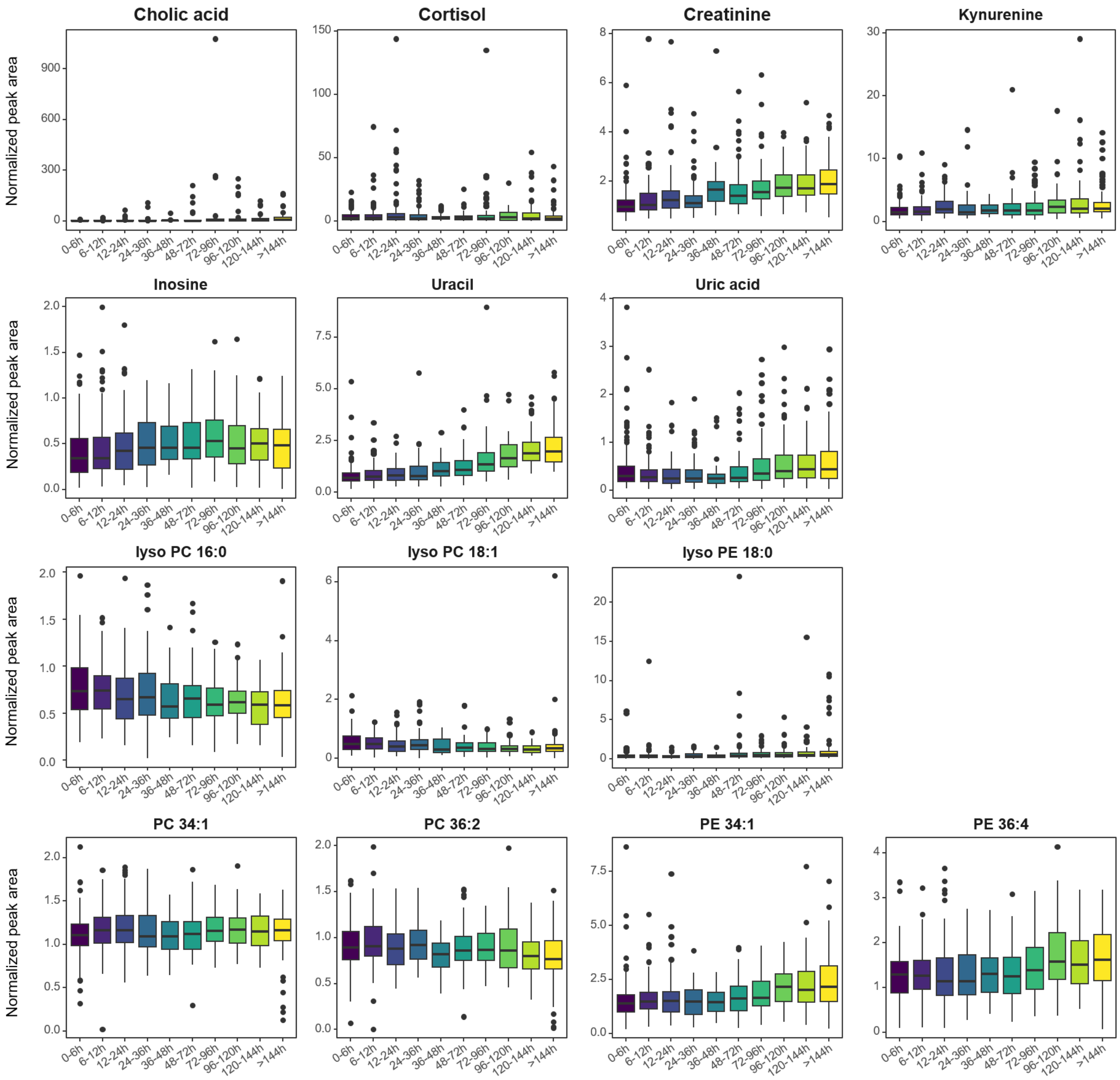

Fig S3: p-value heat map highlighting significant changes determined by Dunn's multiple comparison post-hoc test ($p < 0.05$) between individual postmortem time groups. Light grey areas indicate non-significant results, dark grey to black areas indicate significant changes ($p< 0.05$), with darker areas representing a lower p-value. Figures were created in R.

a: amino acids

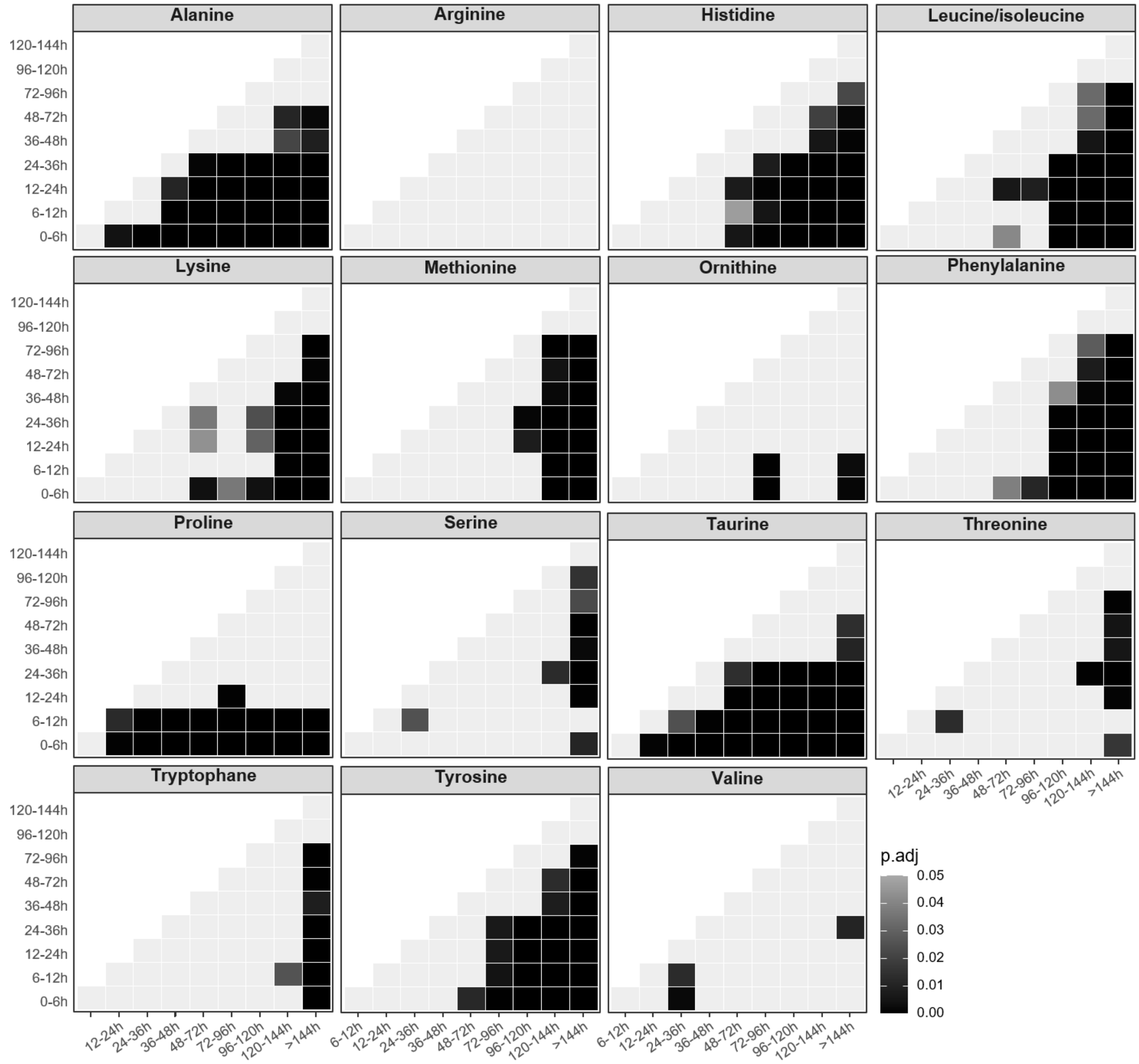

b: carnitines

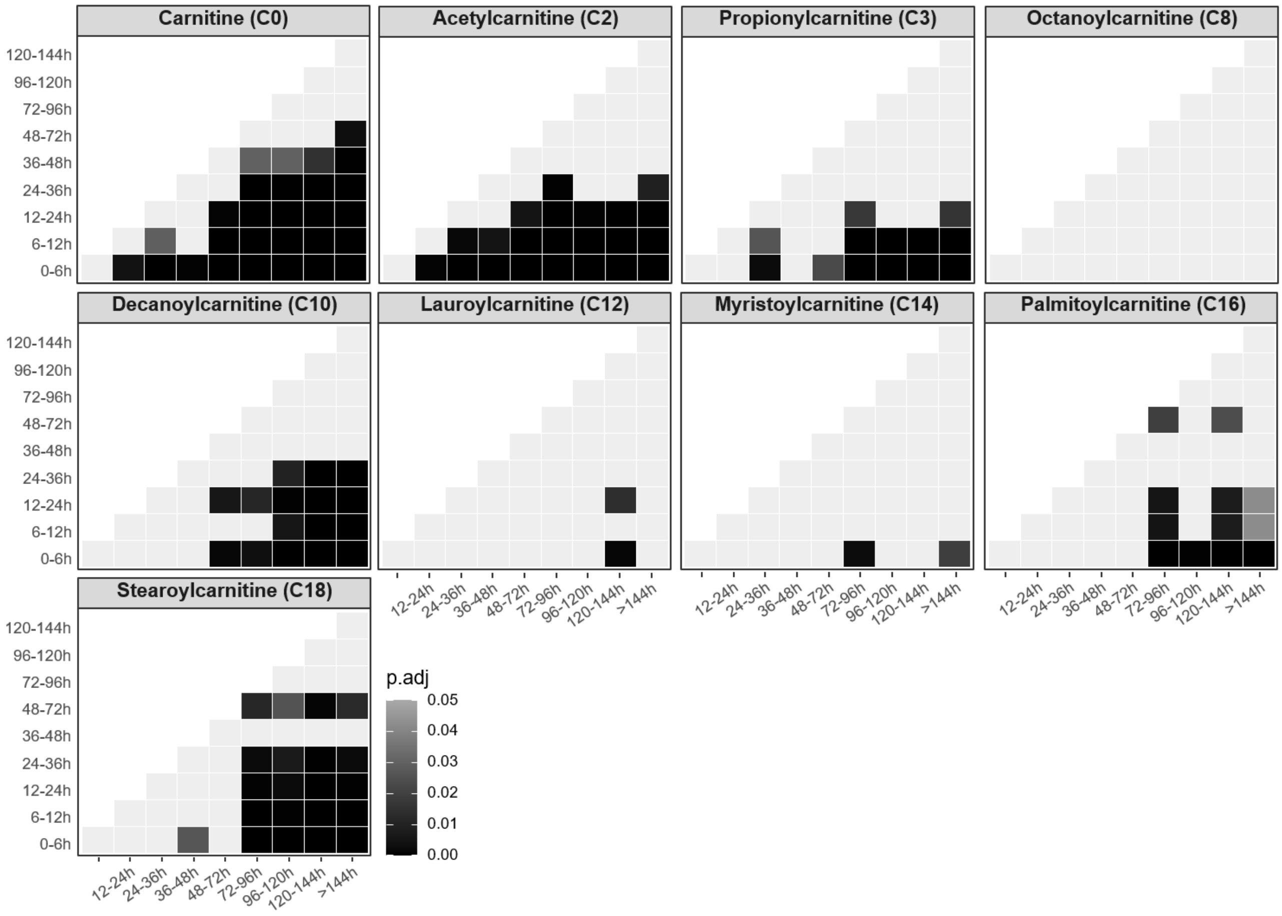

c: other compounds (classes)

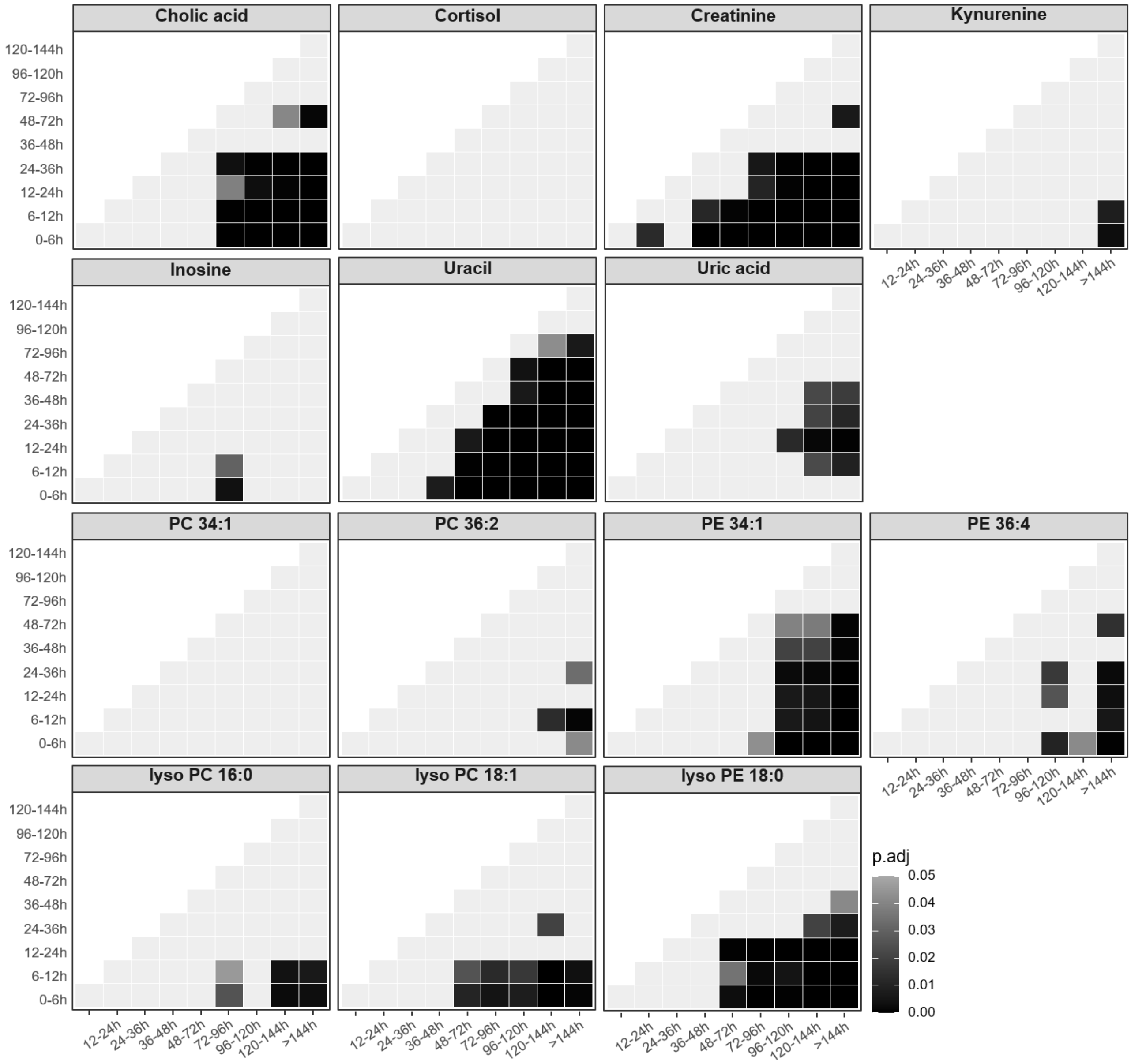

Fig S4: Correlation analysis between median %change between t2 and t1 of each analyte and a) its logP value, b) its molecular weight, c) its retention time in RP chromatography, and d) retention time in HILIC chromatography. Dotted lines indicate no change between paired blood samples. Black squares represent measurement on RP chromatography (n=38 analytes), blue circles measurements in HILIC chromatography (n=18 analytes). GraphPad Prism 10.0.2 was used for figure creation.

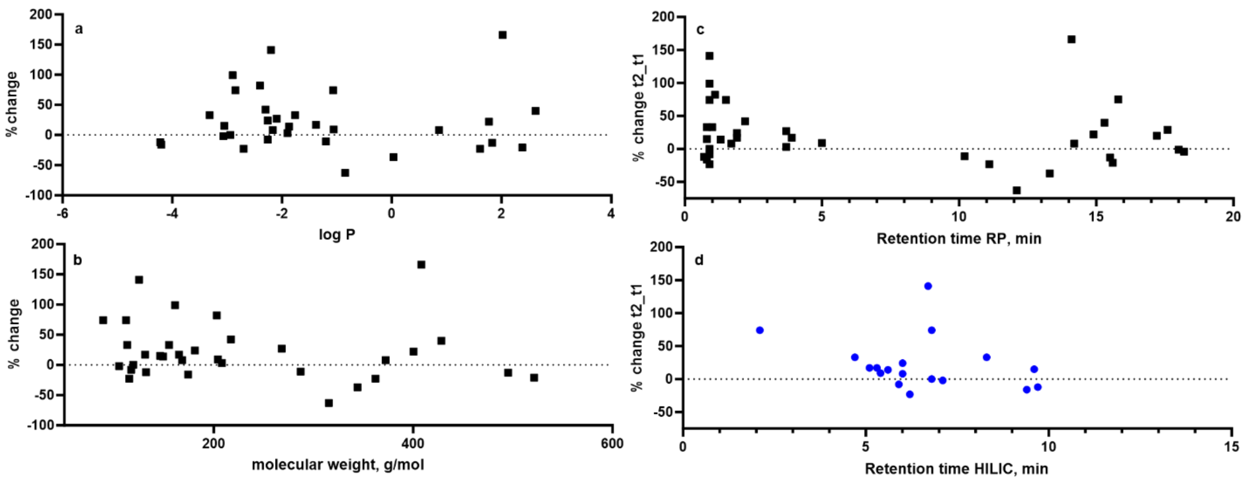

Tables

Table S1: Relative standard deviations (RSD) of absolute peak areas (without normalization) of the internal standards arginine 13C6, creatinine N-methyl D3, and phenylalanine D1and the 38 evaluated analytes in the pool samples and authentic samples measured over 17 batches.

	Pool, RSD (%)	t1, RSD (%)	t2, RSD (%)		Pool, RSD (%)	t1, RSD (%)	t2, RSD (%)
_Arginine 13C6	30	31	26	Leucine	14	49	53
_Creatinine N-Methyl D3	19	30	28	Lysine	16	147	138
_Phenylalanine D1	13	30	30	lyso PC 16:0	15	43	45
Alanine	19	48	34	lyso PC 18:1	15	59	102
Arginine	33	254	220	lyso PE 18:0	39	175	194
C0	12	77	60	Methionine	13	69	78
C10	13	127	161	Ornithine	17	42	54
C12	20	124	130	PC 34:1	24	27	27
C14	23	144	127	PC 36:2	24	31	32
C16	17	81	68	PE 34:1	52	61	60
C18	25	72	58	PE 36:4	45	49	48
C2	11	73	57	Phenylalanine	14	59	59
C3	14	65	65	Proline	15	43	56
C4	13	158	95	Serine	36	61	60
C8	16	123	132	Taurine	20	89	60
Cholic acid	23	299	376	Threonine	36	52	58
Cortisol	15	190	213	Tryptophane	14	67	72
Creatinine	18	65	53	Tyrosine	14	100	89
Histidine	22	110	113	Uracil	15	64	60
Inosine	14	74	58	Uric acid	13	106	103
Isoleucine	14	49	53	Valine	14	46	67
Kynurenine	17	92	111				

Table S2: Median, minimum and maximum changes between paired blood samples (t2_t1) for all cases and according to the time interval between t1 and t2. Statistical testing was performed by Wilcoxon signed-rank test (p<0.05).

		RP					HILIC				
Analyte	Time interval	n	%change median	%change min	%change max	p-value	n	%change median	%change min	%change max	p-value
Alanine	all	427	74	-57	567	***	423	56	-97	892917	***
	0-12h	17	39	-57	229	**	17	10	-85	530	ns
	12-24h	55	39	-27	331	***	54	27	-77	1116	***
	24-36h	26	62	-9	179	***	25	34	-93	4250	ns
	36-48h	50	48	-12	369	***	50	54	-85	3440	***
	48-72h	71	79	-43	281	***	71	67	-90	3767	***
	72-96h	52	89	-23	373	***	51	75	-96	3200	***
	96-120h	48	85	-30	271	***	48	68	-97	1873	***
	120-144h	55	117	-40	406	***	54	67	-96	3978	***
	>144h	54	133	21	567	***	53	91	-96	892917	***
Arginine	all	427	-16	-98	2639	***	422	-9	-100	2085	ns
	0-12h	17	-8	-65	140	ns	17	-17	-59	103	ns
	12-24h	55	1	-61	464	ns	54	14	-56	1036	ns
	24-36h	26	0	-92	341	ns	25	5	-88	450	ns
	36-48h	50	-18	-97	909	ns	50	0	-96	984	ns
	48-72h	71	-14	-98	1173	ns	71	1	-100	1688	ns
	72-96h	52	-15	-91	2639	ns	51	10	-88	2085	ns
	96-120h	48	-32	-92	346	*	48	-19	-97	880	ns
	120-144h	54	-26	-97	1010	ns	54	-30	-100	810	ns
	>144h	54	-45	-98	2619	*	52	-32	-99	1483	**
Histidine	all	427	33	-69	2533	***	420	59	-100	2059427	***
	0-12h	17	18	-51	155	ns	17	11	-61	454632	ns
	12-24h	55	5	-51	159	ns	53	17	-57	614	***
	24-36h	26	16	-46	214	*	25	40	-35	329	**
	36-48h	50	20	-57	255	***	50	47	-66	401	***
	48-72h	71	49	-37	270	***	71	77	-57	646	***

	72-96h	52	30	-41	2533	***	51	71	-62	519	***
	96-120h	48	42	-43	164	***	47	82	-68	1015	***
	120-144h	55	46	-69	248	***	54	61	-87	1146	***
	>144h	54	82	-60	1670	***	52	101	-100	2059427	***
Leucine/isoleucine	all	427	17	-56	635	***	423	35	-91	1786705	***
	0-12h	17	8	-30	126	ns	17	-15	-66	269	ns
	12-24h	55	5	-25	63	*	54	6	-74	995	ns
	24-36h	26	3	-20	85	ns	25	22	-74	682	ns
	36-48h	50	8	-30	331	**	50	31	-90	974	**
	48-72h	71	23	-39	216	***	71	44	-79	1113	***
	72-96h	52	14	-45	635	***	51	38	-88	883	***
	96-120h	48	32	-44	219	***	48	69	-77	1961	***
	120-144h	55	31	-56	216	***	54	41	-91	2906	***
	>144h	54	45	-23	284	***	53	68	-86	1786705	***
Lysine	all	427	15	-80	2638	***	423	20	-97	137263	***
	0-12h	17	6	-36	159	ns	17	-8	-60	192	ns
	12-24h	55	5	-50	95	ns	54	4	-71	474	ns
	24-36h	26	9	-22	77	*	25	13	-66	427	ns
	36-48h	50	7	-70	486	*	50	17	-83	662	**
	48-72h	71	23	-50	358	***	71	39	-73	549	***
	72-96h	52	26	-47	2638	***	51	18	-85	1021	*
	96-120h	48	17	-49	437	**	48	31	-81	1269	**
	120-144h	54	28	-80	307	***	54	24	-97	1947	*
	>144h	54	49	-75	1860	***	53	40	-82	137263	***
Methionine	all	427	14	-62	1850	***	422	33	-85	1631	***
	0-12h	17	10	-37	203	ns	17	-4	-52	209	ns
	12-24h	55	-5	-49	70	ns	54	2	-56	384	ns
	24-36h	26	-6	-24	56	ns	25	15	-51	237	ns
	36-48h	50	-4	-57	348	ns	50	14	-77	713	*
	48-72h	71	14	-54	376	***	71	45	-73	418	***

	72-96h	52	18	-52	1850	**	51	42	-83	839	***
	96-120h	48	39	-62	373	***	48	53	-58	1208	***
	120-144h	55	33	-51	359	***	54	40	-85	1631	**
	>144h	54	53	-27	456	***	52	53	-72	392	***
Ornithine	all	427	-12	-87	471	***	423	-11	-92	40154	***
	0-12h	17	-8	-46	35	ns	17	-18	-62	124	*
	12-24h	55	-9	-74	74	**	54	-4	-83	464	ns
	24-36h	26	-5	-53	77	ns	25	-8	-82	432	ns
	36-48h	50	-13	-59	471	**	50	-2	-90	534	ns
	48-72h	71	-16	-77	163	*	71	-4	-86	598	ns
	72-96h	52	-16	-69	363	*	51	-13	-91	172	ns
	96-120h	48	-17	-78	94	ns	48	-17	-92	728	ns
	120-144h	55	-11	-87	182	ns	54	-16	-89	1147	ns
	>144h	54	-18	-70	395	*	53	-21	-87	40154	**
Phenylalanine	all	427	17	-49	1274	***	423	18	-85	2149900	***
	0-12h	17	9	-37	138	ns	17	-7	-50	156	ns
	12-24h	55	5	-33	61	ns	54	5	-74	479	ns
	24-36h	26	3	-32	77	ns	25	12	-56	400	ns
	36-48h	50	7	-32	286	*	50	11	-83	394	ns
	48-72h	71	18	-41	260	***	71	33	-79	918	**
	72-96h	52	24	-41	1274	***	51	21	-67	1031	*
	96-120h	48	34	-42	263	***	48	39	-75	1119	***
	120-144h	55	31	-49	198	***	54	27	-85	619	*
	>144h	54	57	-23	354	***	53	33	-71	2149900	***
Proline	all	427	-23	-88	847	***	423	17	-92	1307531	***
	0-12h	17	-14	-34	65	*	17	-15	-67	289	ns
	12-24h	55	-23	-52	39	***	54	1	-80	668	ns
	24-36h	26	-25	-50	6	***	25	12	-79	1382	ns
	36-48h	50	-31	-64	140	***	50	19	-89	936	*
	48-72h	71	-30	-67	121	***	71	23	-81	1160	**

	72-96h	52	-34	-71	847	***	51	27	-92	583	*
	96-120h	48	-20	-72	117	**	48	39	-92	1057	***
	120-144h	54	-23	-88	209	***	54	18	-91	2761	ns
	>144h	54	-16	-69	114	**	53	34	-92	1307531	***
Serine	all	426	-2	-81	2536	ns	423	3	-95	145984	**
	0-12h	17	-5	-71	153	ns	17	-22	-79	146	ns
	12-24h	55	-8	-79	259	ns	54	-4	-71	410	ns
	24-36h	26	-16	-72	58	*	25	-14	-91	1204	ns
	36-48h	50	-12	-81	147	*	50	2	-81	1311	ns
	48-72h	70	16	-78	545	ns	71	20	-83	1688	ns
	72-96h	51	7	-65	2536	ns	51	3	-94	737	ns
	96-120h	48	7	-67	340	ns	48	17	-93	525	ns
	120-144h	55	6	-71	270	ns	54	2	-95	2643	ns
	>144h	54	5	-79	297	ns	53	11	-92	145984	*
Threonine	all	427	0	-78	733	ns	423	-5	-94	344133	ns
	0-12h	17	-7	-41	115	ns	17	-15	-75	165	ns
	12-24h	55	-5	-42	60	*	54	-5	-73	307	ns
	24-36h	26	-11	-48	41	*	25	-11	-85	785	ns
	36-48h	50	-9	-50	289	ns	50	-9	-80	1125	ns
	48-72h	71	8	-59	158	ns	71	2	-80	653	ns
	72-96h	52	-1	-50	733	ns	51	0	-94	565	ns
	96-120h	48	12	-55	121	ns	48	6	-92	353	ns
	120-144h	55	11	-63	158	ns	54	-9	-92	1874	ns
	>144h	54	12	-78	161	**	53	-5	-90	344133	ns
Tryptophane	all	427	9	-98	6021	***	422	1	-98	2544	ns
	0-12h	17	1	-33	123	ns	17	-12	-47	189	ns
	12-24h	55	0	-81	70	ns	54	-7	-88	363	ns
	24-36h	26	-4	-35	53	ns	25	-9	-36	175	ns
	36-48h	50	-3	-98	802	ns	50	1	-98	515	ns
	48-72h	71	7	-58	6021	**	71	5	-71	868	ns

	72-96h	52	19	-63	2717	*	51	2	-80	1263	ns
	96-120h	48	37	-91	625	**	48	20	-85	885	ns
	120-144h	55	19	-96	584	**	54	-2	-92	1650	ns
	>144h	54	36	-33	2514	***	52	15	-73	2544	ns
Tyrosine	all	427	24	-97	2656	***	423	37	-97	427133	***
	0-12h	17	14	-28	176	ns	17	11	-43	162	ns
	12-24h	55	6	-31	75	ns	54	11	-76	353	*
	24-36h	26	4	-21	92	ns	25	25	-15	294	***
	36-48h	50	12	-45	538	***	50	32	-67	444	**
	48-72h	71	19	-89	382	***	71	46	-86	498	***
	72-96h	52	42	-36	2656	***	51	51	-70	464	***
	96-120h	48	36	-57	500	***	48	37	-68	1264	***
	120-144h	55	42	-81	494	***	54	42	-79	914	***
	>144h	54	57	-97	561	***	53	46	-97	427133	***
Valine	all	427	-8	-57	811	***	423	45	-94	544252	***
	0-12h	17	-6	-40	26	ns	17	-1	-59	209	ns
	12-24h	55	-11	-43	43	***	54	10	-75	513	*
	24-36h	26	-15	-40	37	*	25	24	-81	1078	*
	36-48h	50	-10	-47	235	**	50	36	-68	1245	***
	48-72h	71	-8	-48	471	ns	71	62	-82	996	***
	72-96h	52	-10	-55	559	ns	51	59	-86	679	***
	96-120h	48	-8	-53	206	ns	48	68	-88	1464	***
	120-144h	55	-7	-57	811	ns	54	51	-94	2602	***
	>144h	54	7	-51	399	ns	53	56	-87	544252	***
Carnitine (C0)	all	427	99	-91	1072	***					
	0-12h	17	34	-75	172	**					
	12-24h	55	45	-27	842	***					
	24-36h	26	80	-22	407	***					
	36-48h	50	56	-66	1072	***					
	48-72h	71	105	-47	530	***					

	72-96h	52	104	-23	814	***					
	96-120h	48	122	-91	670	***					
	120-144h	55	131	-59	860	***					
	>144h	54	212	0	1016	***					
Decanoylcarnitine (C10)	all	419	-63	-100	764	***					
	0-12h	16	-46	-96	60	**					
	12-24h	54	-41	-99	764	***					
	24-36h	26	-34	-94	332	**					
	36-48h	49	-50	-100	343	***					
	48-72h	71	-62	-100	427	***					
	72-96h	52	-67	-100	379	***					
	96-120h	46	-65	-99	207	***					
	120-144h	52	-79	-100	140	***					
	>144h	53	-78	-99	184	***					
Lauroylcarnitine (C12)	all	423	-37	-99	1344	***					
	0-12h	16	-16	-87	1344	*					
	12-24h	55	-25	-97	676	**					
	24-36h	26	-17	-81	144	ns					
	36-48h	49	-24	-97	164	***					
	48-72h	71	-33	-99	600	***					
	72-96h	52	-43	-98	634	***					
	96-120h	48	-48	-99	978	***					
	120-144h	54	-52	-98	177	***					
	>144h	52	-44	-91	158	***					
Myristoylcarnitine (C14)	all	427	8	-93	644	*					
	0-12h	17	8	-78	623	ns					
	12-24h	55	0	-75	310	ns					
	24-36h	26	20	-32	339	ns					
	36-48h	50	1	-81	136	ns					
	48-72h	71	18	-93	644	*					

	72-96h	52	3	-77	330	ns					
	96-120h	48	7	-93	334	ns					
	120-144h	54	3	-72	214	ns					
	>144h	54	20	-87	397	ns					
Palmitoylcarnitine (C16)	all	427	22	-79	566425	***					
	0-12h	17	13	-78	397	ns					
	12-24h	55	-5	-71	435	ns					
	24-36h	26	19	-60	416	ns					
	36-48h	50	-1	-79	204	ns					
	48-72h	71	19	-65	566425	***					
	72-96h	52	29	-55	215	**					
	96-120h	48	28	-73	282	***					
	120-144h	55	30	-42	240	***					
	>144h	54	50	-77	183	***					
Steraroylcarnitine (C18)	all	427	40	-71	657	***					
	0-12h	17	29	-69	178	*					
	12-24h	55	10	-71	186	ns					
	24-36h	26	18	-38	571	*					
	36-48h	50	22	-61	289	**					
	48-72h	71	39	-53	657	***					
	72-96h	52	53	-30	385	***					
	96-120h	48	56	-46	198	***					
	120-144h	55	50	-51	427	***					
	>144h	54	85	-45	313	***					
Acetylcarnitine (C2)	all	424	82	-100	1164	***					
	0-12h	17	35	-15	187	***					
	12-24h	55	61	-43	248	***					
	24-36h	26	95	-28	280	***					
	36-48h	49	77	-66	471	***					
	48-72h	70	106	-92	465	***					

	72-96h	52	88	-100	539	***					
	96-120h	47	83	-70	1164	***					
	120-144h	55	69	-57	975	***					
	>144h	53	109	-40	885	***					
Propionylcarnitine (C3)	all	426	42	-100	525	***					
	0-12h	17	12	-40	181	ns					
	12-24h	55	26	-84	415	***					
	24-36h	26	37	-45	404	**					
	36-48h	50	40	-100	292	***					
	48-72h	70	43	-83	429	***					
	72-96h	52	49	-99	525	***					
	96-120h	48	61	-100	370	***					
	120-144h	55	58	-55	444	***					
	>144h	53	85	-53	380	***					
Octanoylcarnitine (C8)	all	420	-11	-100	9069	**					
	0-12h	17	-32	-100	9069	ns					
	12-24h	55	-13	-99	1703	ns					
	24-36h	26	13	-92	509	ns					
	36-48h	48	-8	-99	1815	ns					
	48-72h	70	6	-99	1127	ns					
	72-96h	51	-18	-98	704	ns					
	96-120h	45	13	-98	1604	ns					
	120-144h	54	-17	-100	581	*					
	>144h	54	-29	-97	1244	ns					
Cholic acid	all	240	166	-76	74485	***					
	0-12h	9	-6	-45	25373	ns					
	12-24h	24	-6	-58	485	ns					
	24-36h	17	45	-76	5412	ns					
	36-48h	24	35	-52	4775	**					
	48-72h	49	77	-64	20432	**					

	72-96h	31	375	-67	74485	***					
	96-120h	25	286	-49	24368	***					
	120-144h	29	1228	-18	22187	***					
	>144h	32	903	-15	41107	***					
Cortisol	all	423	-23	-87	195	***					
	0-12h	17	-8	-39	159	ns					
	12-24h	54	-8	-56	62	ns					
	24-36h	26	-6	-43	70	ns					
	36-48h	49	-16	-60	123	***					
	48-72h	71	-23	-64	195	**					
	72-96h	51	-25	-80	35	***					
	96-120h	47	-21	-87	100	***					
	120-144h	54	-27	-79	31	***					
	>144h	54	-38	-86	28	***					
Creatinine	all	427	33	-39	509	***	423	73	-90	752294	***
	0-12h	17	6	-22	52	ns	17	11	-67	156	ns
	12-24h	55	14	-16	80	***	54	26	-70	695	***
	24-36h	26	20	-33	121	*	25	37	-68	1798	*
	36-48h	50	23	-36	168	***	50	59	-71	1174	***
	48-72h	71	43	-25	509	***	71	99	-74	1941	***
	72-96h	52	39	-17	171	***	51	84	-86	1630	***
	96-120h	48	44	-24	251	***	48	95	-87	979	***
	120-144h	54	43	-39	254	***	54	77	-90	2545	***
	>144h	54	70	-21	226	***	53	119	-90	752294	***
Kynurenine	all	371	3	-71	1771	**					
	0-12h	13	-12	-71	210	ns					
	12-24h	44	-5	-62	43	ns					
	24-36h	23	-3	-35	77	ns					
	36-48h	45	1	-43	106	ns					
	48-72h	64	6	-63	267	*					

	72-96h	46	0	-60	453	ns					
	96-120h	42	3	-57	1771	ns					
	120-144h	48	11	-63	869	**					
	>144h	46	21	-31	979	**					
Taurine	all	426	141	-73	2389	***	423	89	-92	591146	***
	0-12h	17	68	-46	199	***	17	19	-68	168	ns
	12-24h	55	93	-25	707	***	54	56	-62	1073	***
	24-36h	26	112	-42	351	***	25	61	-82	2397	**
	36-48h	50	118	-17	793	***	50	72	-79	1370	***
	48-72h	71	154	-43	989	***	71	96	-69	1880	***
	72-96h	50	162	-66	532	***	51	75	-83	1589	***
	96-120h	48	158	-73	807	***	48	118	-92	1367	***
	120-144h	55	189	-50	787	***	54	96	-88	2213	***
	>144h	54	247	11	2389	***	53	117	-90	591146	***
Inosine	all	381	27	-99	1989	***					
	0-12h	17	9	-86	427	ns					
	12-24h	54	28	-86	537	ns					
	24-36h	26	37	-36	397	**					
	36-48h	46	27	-78	392	**					
	48-72h	62	42	-94	1654	*					
	72-96h	44	26	-92	1956	**					
	96-120h	40	8	-89	613	ns					
	120-144h	44	19	-96	1989	ns					
	>144h	48	27	-99	1492	ns					
Uracil	all	427	74	-52	760	***	423	50	-99	3538586	***
	0-12h	17	38	-39	127	***	17	-10	-64	148	ns
	12-24h	55	29	-52	113	***	54	26	-76	594	*
	24-36h	26	47	-17	287	***	25	33	-81	1379	**
	36-48h	50	54	-25	283	***	50	41	-73	1636	***
	48-72h	71	56	-49	513	***	71	52	-81	1592	***

	72-96h	52	92	-12	419	***	51	58	-86	1435	***
	96-120h	48	101	-33	547	***	48	97	-92	2002	***
	120-144h	54	146	-39	560	***	54	81	-99	2900	***
	>144h	54	161	3	760	***	53	114	-87	3538586	***
Uric acid	all	392	8	-97	4351	***	422	4	-100	4119297	**
	0-12h	17	-9	-86	155	ns	17	-15	-68	48	*
	12-24h	51	-6	-65	754	ns	54	-7	-100	524	ns
	24-36h	26	-10	-85	350	ns	25	-9	-75	502	ns
	36-48h	44	11	-97	840	ns	50	1	-100	1091301	ns
	48-72h	66	-3	-89	4351	ns	71	17	-100	965715	ns
	72-96h	47	0	-92	778	ns	51	4	-99	133321	ns
	96-120h	44	29	-64	1333	***	48	32	-100	200575	**
	120-144h	51	29	-61	1659	**	53	31	-99	158169	*
	>144h	46	38	-81	2558	**	53	8	-100	4119297	ns
lyso PC 16:0	all	427	-13	-87	227	***					
	0-12h	17	-13	-60	145	ns					
	12-24h	55	-9	-64	119	*					
	24-36h	26	-9	-78	76	ns					
	36-48h	50	-16	-82	123	**					
	48-72h	71	-14	-82	225	**					
	72-96h	52	-1	-70	227	ns					
	96-120h	48	-15	-57	112	*					
	120-144h	55	-14	-82	86	***					
	>144h	54	-11	-87	94	*					
lyso PC 18:1	all	427	-21	-93	1020	***					
	0-12h	17	-18	-79	182	ns					
	12-24h	55	-20	-78	228	*					
	24-36h	26	-20	-91	178	ns					
	36-48h	50	-27	-93	198	**					
	48-72h	71	-25	-93	277	***					

	72-96h	52	-10	-85	311	*					
	96-120h	48	-22	-70	136	***					
	120-144h	55	-21	-88	518	***					
	>144h	54	-25	-90	1020	**					
lyso PE 18:0	all	427	75	-81	3758	***					
	0-12h	17	36	-72	264	ns					
	12-24h	55	15	-80	945	ns					
	24-36h	26	64	-65	566	*					
	36-48h	50	45	-62	1062	**					
	48-72h	71	112	-72	2488	***					
	72-96h	52	120	-81	1258	***					
	96-120h	47	71	-74	1478	***					
	120-144h	55	80	-59	3758	***					
	>144h	54	125	-78	2092	***					
PC 34:1	all	427	-1	-81	494	ns					
	0-12h	17	1	-52	138	ns					
	12-24h	55	1	-49	70	ns					
	24-36h	26	-4	-47	54	ns					
	36-48h	50	0	-36	34	ns					
	48-72h	71	-4	-47	113	ns					
	72-96h	52	4	-27	140	ns					
	96-120h	48	-2	-57	102	ns					
	120-144h	55	1	-81	494	ns					
	>144h	54	0	-80	128	ns					
PC 36:2	all	427	-4	-93	359	***					
	0-12h	17	3	-49	134	ns					
	12-24h	55	1	-55	45	ns					
	24-36h	26	-6	-47	69	ns					
	36-48h	50	-7	-48	44	**					
	48-72h	71	-2	-60	116	ns					

	72-96h	52	-4	-30	75	ns						
	96-120h	48	-5	-61	184	*						
	120-144h	55	-10	-93	359	*						
	>144h	54	1	-92	321	ns						
PE 34:1	all	420	29	-85	652	***						
	0-12h	17	12	-67	106	ns						
	12-24h	55	2	-69	138	ns						
	24-36h	26	14	-42	182	ns						
	36-48h	50	9	-55	243	**						
	48-72h	67	38	-78	560	*						
	72-96h	51	36	-56	652	***						
	96-120h	46	54	-69	585	***						
	120-144h	55	57	-85	526	***						
	>144h	53	36	-64	338	***						
PE 36:4	all	427	20	-95	1865	***						
	0-12h	17	10	-58	124	ns						
	12-24h	55	3	-72	179	ns						
	24-36h	26	13	-59	180	ns						
	36-48h	50	6	-67	145	ns						
	48-72h	71	17	-66	1865	**						
	72-96h	52	28	-51	544	***						
	96-120h	48	24	-60	916	**						
	120-144h	55	40	-95	168	***						
	>144h	54	37	-86	903	***						

Table S3: Median percent changes between time groups to group 1 (0-6h). Statistical testing was performed by Kruskal-Wallis test (p<0.05) followed by Dunn's multiple comparison test of significant features.

		RP				HILIC			
Analyte	Group	n	median %change to time group 0-6h	p-value Kruskal-Wallis	adj. p-value Dunn's	n	median %change to time group 0-6h	p-value Krukal-Wallis	adj. p-value Dunn's
Alanine				***				***	
	0-6h	150	0			150	0		
	6-12h	141	22		ns	139	7		ns
	12-24h	94	35		**	94	13		ns
	24-36h	78	58		***	77	23		ns
	36-48h	34	113		***	34	38		ns
	48-72h	79	104		***	79	59		***
	72-96h	75	140		***	74	91		***
	96-120h	47	144		***	47	84		***
	120-144h	58	164		***	57	106		***
	>144h	99	172		***	99	102		***
Arginine				ns				ns	
	0-6h	150	0			150	0		
	6-12h	141	17			139	16		
	12-24h	94	27			93	31		
	24-36h	78	25			77	20		
	36-48h	34	25			34	18		
	48-72h	79	0			79	19		
	72-96h	75	18			74	11		
	96-120h	47	13			47	13		
	120-144h	58	-19			57	-12		
	>144h	98	-21			99	-16		
Histidine				***				***	
	0-6h	150	0			150	0		
	6-12h	141	3		ns	139	-1		ns

	12-24h	94	0		ns	93	9		ns
	24-36h	78	-3		ns	76	19		ns
	36-48h	34	11		ns	34	44		ns
	48-72h	79	26		**	79	37		***
	72-96h	75	30		***	74	65		***
	96-120h	47	29		***	47	58		***
	120-144h	58	56		***	57	79		***
	>144h	99	71		***	98	107		***
Leucine / isoleucine				***				***	
	0-6h	150	0			150	0		
	6-12h	141	2		ns	139	-5		ns
	12-24h	94	-3		ns	94	-8		ns
	24-36h	78	-1		ns	77	-1		ns
	36-48h	34	7		ns	34	-1		ns
	48-72h	79	17		*	79	8		ns
	72-96h	75	10		ns	74	12		ns
	96-120h	47	29		***	47	27		ns
	120-144h	58	28		***	57	45		***
	>144h	99	51		***	99	59		***
Lysine				***				***	
	0-6h	150	0			150	0		
	6-12h	141	10		ns	139	-4		ns
	12-24h	94	2		ns	94	-6		ns
	24-36h	78	0		ns	77	-2		ns
	36-48h	34	6		ns	34	-6		ns
	48-72h	79	18		**	79	8		ns
	72-96h	75	17		*	74	16		ns
	96-120h	47	22		**	47	15		ns
	120-144h	57	54		***	57	39		**
	>144h	99	57		***	99	50		***

Methionine				***				***	
	0-6h	150	0			150	0		
	6-12h	141	1		ns	139	6		ns
	12-24h	94	-5		ns	93	0		ns
	24-36h	78	-10		ns	77	9		ns
	36-48h	34	-3		ns	34	16		ns
	48-72h	79	2		ns	79	21		ns
	72-96h	75	-8		ns	74	25		ns
	96-120h	47	13		ns	47	30		*
	120-144h	58	36		***	57	65		***
	>144h	99	45		***	99	72		***
Ornithine				***				**	
	0-6h	150	0			150	0		
	6-12h	141	-4		ns	139	-11		ns
	12-24h	94	-14		ns	94	-17		ns
	24-36h	78	-11		ns	77	-14		ns
	36-48h	34	-6		ns	34	-21		ns
	48-72h	79	-14		ns	79	-16		ns
	72-96h	75	-25		***	74	-25		**
	96-120h	47	-18		ns	47	-27		ns
	120-144h	58	-9		ns	57	-21		ns
	>144h	99	-23		**	99	-31		*
Phenylalanine				***				***	
	0-6h	150	0			150	0		
	6-12h	141	2		ns	139	-11		ns
	12-24h	94	3		ns	94	-8		ns
	24-36h	78	4		ns	77	-8		ns
	36-48h	34	10		ns	34	-4		ns
	48-72h	79	15		*	79	-5		ns
	72-96h	75	18		*	74	7		ns

	96-120h	47	30		***	47	15		ns
	120-144h	58	44		***	57	22		*
	>144h	99	59		***	99	35		***
Proline				***				***	
	0-6h	150	0			150	0		
	6-12h	141	-6		ns	139	-3		ns
	12-24h	94	-25		***	94	-3		ns
	24-36h	78	-31		***	77	-6		ns
	36-48h	34	-34		***	34	-4		ns
	48-72h	79	-35		***	79	6		ns
	72-96h	75	-40		***	74	14		ns
	96-120h	47	-41		***	47	14		ns
	120-144h	58	-33		***	57	28		*
	>144h	98	-34		***	99	39		***
Serine				***				***	
	0-6h	149	0			150	0		
	6-12h	141	1		ns	139	-8		ns
	12-24h	94	-8		ns	94	-17		ns
	24-36h	78	-19		ns	77	-12		ns
	36-48h	34	-17		ns	34	-18		ns
	48-72h	78	-9		ns	79	-9		ns
	72-96h	74	-3		ns	74	-3		ns
	96-120h	47	-7		ns	47	-1		ns
	120-144h	58	9		ns	57	8		ns
	>144h	99	23		*	99	22		ns
Threonine				***				***	
	0-6h	150	0			150	0		
	6-12h	141	-1		ns	139	-10		ns
	12-24h	94	-7		ns	94	-16		ns
	24-36h	78	-15		ns	77	-20		ns

	36-48h	34	-14		ns	34	-25		ns
	48-72h	79	-9		ns	79	-20		ns
	72-96h	75	-10		ns	74	-19		ns
	96-120h	47	-2		ns	47	-16		ns
	120-144h	58	7		ns	57	-7		ns
	>144h	99	11		*	99	-2		ns
Tryptophane				***				***	
	0-6h	150	0			150	0		
	6-12h	141	-3		ns	139	-17		*
	12-24h	94	2		ns	93	-18		ns
	24-36h	78	0		ns	77	-14		ns
	36-48h	34	5		ns	34	-20		ns
	48-72h	79	5		ns	79	-13		ns
	72-96h	75	3		ns	74	-12		ns
	96-120h	47	19		ns	47	-12		ns
	120-144h	58	23		ns	57	-3		ns
	>144h	99	42		***	99	4		ns
Tyrosine				***				***	
	0-6h	150	0			150	0		
	6-12h	141	5		ns	139	-1		ns
	12-24h	94	2		ns	94	5		ns
	24-36h	78	0		ns	77	10		ns
	36-48h	34	11		ns	34	23		ns
	48-72h	79	22		*	79	25		**
	72-96h	75	26		***	74	45		***
	96-120h	47	36		***	47	46		***
	120-144h	58	50		***	57	71		***
	>144h	99	79		***	99	78		***
Valine				***				***	
	0-6h	150	0			150	0		

	6-12h	141	0		ns	139	-2		ns
	12-24h	94	-6		ns	94	0		ns
	24-36h	78	-14		**	77	12		ns
	36-48h	34	-13		ns	34	5		ns
	48-72h	79	-13		ns	79	19		*
	72-96h	75	-11		ns	74	41		***
	96-120h	47	-11		ns	47	33		**
	120-144h	58	-3		ns	57	110		***
	>144h	99	1		ns	99	92		***
Carnitine (C0)				***					
	0-6h	150	0						
	6-12h	141	33		ns				
	12-24h	94	58		**				
	24-36h	78	92		***				
	36-48h	34	123		***				
	48-72h	79	139		***				
	72-96h	75	224		***				
	96-120h	47	237		***				
	120-144h	58	234		***				
	>144h	99	274		***				
Decanoylcarnitine (C10)				***					
	0-6h	149	0						
	6-12h	141	-21		ns				
	12-24h	94	16		ns				
	24-36h	77	-15		ns				
	36-48h	34	-46		ns				
	48-72h	78	-50		**				
	72-96h	75	-56		**				
	96-120h	47	-66		***				
	120-144h	55	-75		***				

	>144h	96	-80		***				
Lauroylcarnitine (C12)				**					
	0-6h	149	0						
	6-12h	141	-13		ns				
	12-24h	94	-13		ns				
	24-36h	78	-14		ns				
	36-48h	34	-20		ns				
	48-72h	78	-23		ns				
	72-96h	75	-31		ns				
	96-120h	47	-21		ns				
	120-144h	58	-54		**				
	>144h	96	-42		ns				
Myristoylcarnitine (C14)				**					
	0-6h	150	0						
	6-12h	141	19		ns				
	12-24h	94	29		ns				
	24-36h	78	39		ns				
	36-48h	34	27		ns				
	48-72h	79	44		ns				
	72-96h	75	73		**				
	96-120h	47	54		ns				
	120-144h	58	51		ns				
	>144h	98	49		*				
Palmitoylcarnitine (C16)				***					
	0-6h	150	0						
	6-12h	141	7		ns				
	12-24h	94	12		ns				
	24-36h	78	19		ns				
	36-48h	34	31		ns				
	48-72h	79	17		ns				

	72-96h	75	70		***				
	96-120h	47	60		***				
	120-144h	58	70		***				
	>144h	99	43		***				
Steraroylcarnitine (C18)				***					
	0-6h	150	0						
	6-12h	141	24		ns				
	12-24h	94	29		ns				
	24-36h	78	38		ns				
	36-48h	34	64		*				
	48-72h	79	46		ns				
	72-96h	75	97		***				
	96-120h	47	98		***				
	120-144h	58	123		***				
	>144h	99	85		***				
Acetylcarnitine (C2)				***					
	0-6h	150	0						
	6-12h	140	27		ns				
	12-24h	94	61		***				
	24-36h	78	94		***				
	36-48h	34	111		***				
	48-72h	77	121		***				
	72-96h	75	227		***				
	96-120h	47	178		***				
	120-144h	57	154		***				
	>144h	98	175		***				
Propionylcarnitine (C3)				***					
	0-6h	150	0						
	6-12h	140	6		ns				
	12-24h	94	31		ns				

	24-36h	78	65		**				
	36-48h	34	40		ns				
	48-72h	78	44		*				
	72-96h	75	98		***				
	96-120h	47	90		***				
	120-144h	58	94		***				
	>144h	98	90		***				
Octanoylcarnitine (C8)				ns					
	0-6h	150	0						
	6-12h	141	8						
	12-24h	94	28						
	24-36h	78	27						
	36-48h	34	14						
	48-72h	76	0						
	72-96h	75	35						
	96-120h	45	33						
	120-144h	55	-1						
	>144h	99	-1						
Cholic acid				***					
	0-6h	78	0						
	6-12h	83	24		ns				
	12-24h	61	49		ns				
	24-36h	45	22		ns				
	36-48h	19	198		ns				
	48-72h	55	92		ns				
	72-96h	59	399		***				
	96-120h	41	524		***				
	120-144h	53	862		***				
	>144h	89	1190		***				
Cortisol				ns					

	0-6h	150	0						
	6-12h	138	14						
	12-24h	94	30						
	24-36h	77	-10						
	36-48h	34	-12						
	48-72h	77	-1						
	72-96h	73	-4						
	96-120h	47	27						
	120-144h	57	-15						
	>144h	98	-12						
Creatinine				***				***	
	0-6h	150	0			150	0		
	6-12h	140	9		ns	139	12		ns
	12-24h	94	28		*	94	24		ns
	24-36h	78	17		ns	77	28		ns
	36-48h	34	73		***	34	90		**
	48-72h	79	47		***	79	63		***
	72-96h	75	63		***	74	123		***
	96-120h	47	80		***	47	101		***
	120-144h	58	79		***	57	161		***
	>144h	98	97		***	99	179		***
Kynurenine				***					
	0-6h	131	0						
	6-12h	122	6		ns				
	12-24h	79	37		ns				
	24-36h	68	2		ns				
	36-48h	32	22		ns				
	48-72h	70	25		ns				
	72-96h	70	24		ns				
	96-120h	43	62		ns				

	120-144h	54	41		ns				
	>144h	90	42		**				
Taurine				***				***	
	0-6h	148	0			150	0		
	6-12h	141	36		ns	139	13		ns
	12-24h	94	95		***	94	43		ns
	24-36h	78	130		***	77	70		**
	36-48h	34	216		***	34	129		**
	48-72h	79	230		***	79	135		***
	72-96h	75	367		***	74	172		***
	96-120h	47	332		***	47	169		***
	120-144h	58	379		***	57	211		***
	>144h	99	361		***	99	215		***
Inosine				***					
	0-6h	140	0						
	6-12h	136	0		ns				
	12-24h	91	23		ns				
	24-36h	76	32		ns				
	36-48h	33	32		ns				
	48-72h	75	33		ns				
	72-96h	68	54		**				
	96-120h	41	30		ns				
	120-144h	50	47		ns				
	>144h	87	41		ns				
Uracil				***				***	
	0-6h	150	0			150	0		
	6-12h	141	6		ns	139	7		ns
	12-24h	94	16		ns	94	5		ns
	24-36h	78	12		ns	77	15		ns
	36-48h	34	46		**	34	23		ns

	48-72h	79	53		***	79	31		ns
	72-96h	75	89		***	74	58		***
	96-120h	47	132		***	47	77		***
	120-144h	58	166		***	57	87		***
	>144h	98	176		***	99	108		***
Uric acid				***				***	
	0-6h	150	0			150	0		
	6-12h	134	-6		ns	139	-21		ns
	12-24h	88	-16		ns	94	-24		*
	24-36h	75	-15		ns	77	-20		ns
	36-48h	33	-17		ns	34	-31		ns
	48-72h	73	-12		ns	79	-13		ns
	72-96h	70	22		ns	74	13		ns
	96-120h	46	35		ns	47	2		ns
	120-144h	56	49		ns	57	13		ns
	>144h	94	49		ns	98	20		ns
lyso PC 16:0				***					
	0-6h	150	0						
	6-12h	141	0		ns				
	12-24h	94	-11		ns				
	24-36h	78	-9		ns				
	36-48h	34	-22		ns				
	48-72h	79	-11		ns				
	72-96h	75	-20		*				
	96-120h	47	-16		ns				
	120-144h	58	-20		**				
	>144h	99	-20		**				
lyso PC 18:1				***					
	0-6h	150	0						
	6-12h	141	0		ns				

	12-24h	94	-15		ns				
	24-36h	78	-8		ns				
	36-48h	34	-36		ns				
	48-72h	79	-25		*				
	72-96h	75	-32		**				
	96-120h	47	-35		**				
	120-144h	58	-38		***				
	>144h	99	-28		***				
lyso PE 18:0				***					
	0-6h	149	0						
	6-12h	141	3		ns				
	12-24h	94	-5		ns				
	24-36h	78	23		ns				
	36-48h	34	9		ns				
	48-72h	79	52		**				
	72-96h	75	78		***				
	96-120h	47	76		***				
	120-144h	58	93		***				
	>144h	99	104		***				
PC 34:1				ns					
	0-6h	150	0						
	6-12h	141	5						
	12-24h	94	5						
	24-36h	78	-1						
	36-48h	34	-2						
	48-72h	79	1						
	72-96h	75	5						
	96-120h	47	6						
	120-144h	58	4						
	>144h	99	5						

PC 36:2				***					
	0-6h	150	0						
	6-12h	141	1		ns				
	12-24h	94	-2		ns				
	24-36h	78	3		ns				
	36-48h	34	-9		ns				
	48-72h	79	-4		ns				
	72-96h	75	-3		ns				
	96-120h	47	-4		ns				
	120-144h	58	-10		ns				
	>144h	99	-15		*				
PE 34:1				***					
	0-6h	149	0						
	6-12h	138	6		ns				
	12-24h	91	7		ns				
	24-36h	77	6		ns				
	36-48h	34	2		ns				
	48-72h	79	16		ns				
	72-96h	74	18		*				
	96-120h	47	53		***				
	120-144h	58	44		***				
	>144h	99	53		***				
PE 36:4				***					
	0-6h	150	0						
	6-12h	141	-1		ns				
	12-24h	94	-11		ns				
	24-36h	78	-11		ns				
	36-48h	34	2		ns				
	48-72h	79	-3		ns				
	72-96h	75	8		ns				

	96-120h	47	23		**				
	120-144h	58	18		*				
	>144h	99	26		***				
